# Prevalence of Chronic Respiratory Diseases, Airflow Obstruction, Sleep Apnea Risk, and Novel Tobacco Use in the Coastal Localities of the Mangalia Municipality: A Cross-Sectional Study of 979 Adults

**DOI:** 10.3390/life16101666

**Published:** 2026-10-04

**Authors:** Florin Dumitru Mihălțan, Andrei-Antonio Cotea, Andreea-Nicoleta Mălăescu, Ancuța-Alina Constantin

**Affiliations:** 1 Department of Cardio-Thoracic Pathology, “Carol Davila” University of Medicine and Pharmacy, 050474 Bucharest, Romania; florin.mihaltan@umfcd.ro (F.D.M.); andreea-nicoleta.malaescu@rez.umfcd.ro (A.-N.M.); ancuta-alina.constantin@umfcd.ro (A.-A.C.); 2 Institute of Pneumology “Marius Nasta”, 050159 Bucharest, Romania; 3 American Thoracic Society International Health Committee, 25 Broadway, New York, NY 10004, USA

**Keywords:** chronic respiratory disease, airflow obstruction, community-based screening, obstructive sleep apnea risk, novel tobacco products, COPD

## Abstract

Background: Spirometric airflow obstruction frequently remains unrecognised in community settings, representing a major diagnostic gap in chronic respiratory disease management. Population-based epidemiological data from Romanian coastal communities–characterised by a highly dynamic population including residents, seasonal workers, and tourists–remain scarce. This study aimed to estimate the prevalence of screening-detected pre-bronchodilator airflow obstruction and chronic respiratory diseases, characterise smoking behaviours including novel tobacco product use, identify individuals at elevated risk for sleep apnea, and examine patterns of preventive care and healthcare utilisation across five Black Sea coastal localities. Methods: A cross-sectional survey was conducted among 979 adults from five coastal localities in Constanța County, Romania, as part of a community-based respiratory screening campaign conducted 11–16 August 2025: Neptun (*n* = 147), Olimp (*n* = 178), Saturn (*n* = 187), Mangalia (*n* = 257), and Jupiter (*n* = 210). Data were collected using a structured questionnaire covering 22 domains, including medical diagnoses, spirometric measurements (FEV1/FVC%), sleep quality, snoring, anthropometric parameters, comorbidities, and healthcare utilisation. Airflow obstruction was defined as an FEV1/FVC ratio < 70%. The primary outcome of interest was the prevalence of screening-detected pre-bronchodilator airflow obstruction, including among individuals without a prior respiratory diagnosis. Sleep apnea risk was defined by the co-reporting of habitual snoring and witnessed apneic episodes, used as a two-item screening proxy. Results: Among 979 participants (51.4% female; mean age 47.3 ± 13.5 years), 9.3% had at least one CRD: asthma (4.4%), chronic bronchitis (3.0%), COPD (1.7%), and emphysema (0.3%). CRD prevalence increased with age, from 6.1% in individuals <40 years to 24.2% in those aged 71–80 years. Of 973 participants with spirometry data, 17.9% demonstrated airflow obstruction (FEV_1_/FVC < 70%), including 15.7% without a prior respiratory diagnosis, representing previously unrecognised airflow limitation rather than confirmed chronic disease. Screening-defined OSA-risk positivity, based on a two-item proxy of habitual snoring and witnessed apnoeic episodes, was identified in 10.9% (*n* = 107) and was more common among obese participants (19.5% vs. 7.9%; absolute difference 11.6 percentage points; *p* < 0.001). Current smoking was reported by 35.2%, and 8.9% reported the use of novel tobacco products. Influenza vaccination coverage was 20.0%, while pneumococcal vaccination uptake was 3.4%. Conclusions: One in six adults without a prior respiratory diagnosis demonstrated spirometric airflow limitation, suggesting that opportunistic spirometry in primary care could substantially improve case detection in Romanian coastal communities. OSA risk, assessed by symptom-based screening proxy, was present in 10.9% of participants, with higher BMI as the strongest independent predictor among the variables assessed. High smoking prevalence (35.2%) and critically low pneumococcal vaccination coverage (3.4%) represent additional modifiable public health targets. These findings support a unified community respiratory screening approach incorporating spirometry, sleep apnea risk stratification, and vaccination audit, particularly in primary care settings serving coastal populations with dynamic demographics.

## 1. Introduction

Chronic respiratory diseases–encompassing chronic obstructive pulmonary disease (COPD), asthma, pneumoconiosis, interstitial lung disease, and pulmonary sarcoidosis–constitute a leading cause of morbidity and mortality worldwide [1]. In 2019, CRDs ranked as the third leading cause of death globally, responsible for approximately 4.0 million deaths and 454.6 million prevalent cases; by 2023, the number of prevalent cases had risen further to 569.2 million, despite declines in age-standardised rates reflecting improvements in population health [2,3].

Chronic obstructive pulmonary disease is a preventable condition characterised by persistent, partially irreversible airflow limitation (FEV_1_/FVC < 70%) resulting from progressive lower airway inflammation, with tobacco smoking as the principal–though not exclusive–risk factor [4]. It encompasses a spectrum of pathological entities including chronic bronchitis with fixed airway obstruction, emphysema (centrilobular forms typically linked to smoking; panlobular forms to α_1_-antitrypsin deficiency), and advanced stages complicated by chronic hypoxaemic respiratory failure [5]. In 2015, COPD affected an estimated 174.5 million individuals worldwide and was responsible for approximately 3.2 million deaths. Although COPD mortality has declined across Europe–particularly among men–it remains a major contributor to healthcare burden and costs, with this downward trend not uniform across all European countries [6].

In Romania, the Romanian Society of Pneumology conducted national COPD surveys between 2012 and 2018 and published a national clinical guideline for COPD diagnosis and management in 2019. COPD diagnosis rests on three key components: relevant exposure history, respiratory symptoms, and persistent, not fully reversible airflow limitation, with further assessment of symptom burden, exacerbation frequency, comorbidities, and disease phenotypes [7]. Robust epidemiological data on disease prevalence, severity, and risk factors are critical for informing effective public health and clinical interventions [4].

Asthma is a heterogeneous chronic airway disease driven by underlying airway inflammation and defined clinically by variable respiratory symptoms–wheeze, dyspnoea, chest tightness, and cough–accompanied by variable expiratory airflow limitation that may become persistent as the disease progresses. Airway hyperresponsiveness frequently accompanies this process, though it is neither required nor sufficient alone to confirm the diagnosis. Unlike COPD, asthma is not primarily smoking-driven; its allergic phenotype, the most readily recognised form, is instead linked to atopic sensitisation and a personal or family history of allergic disease, while a distinct non-allergic phenotype exists independently of atopy [8]. In the present cohort, asthma constituted the single most prevalent CRD subtype (4.4%), underscoring its relevance to the overall findings.

Obstructive sleep apnea (OSA) is characterised by recurrent episodes of partial or complete upper airway obstruction during sleep, resulting in reduced airflow despite ongoing respiratory effort. These events are typically associated with intermittent oxygen desaturation and cortical microarousals, leading to sleep fragmentation and increased sympathetic activity [9]. Obesity is a chronic medical condition defined by excessive body fat accumulation that impairs health, commonly assessed using body mass index (BMI), with values ≥30 kg/m^2^ classified as obese. It is a major risk factor for OSA, primarily through anatomical and mechanical mechanisms: increased fat deposition in the upper airway promotes pharyngeal narrowing and collapsibility during sleep, while excess abdominal mass elevates diaphragmatic pressure and reduces lung volumes, together increasing the likelihood of recurrent airway obstruction and apnoeic events [10]. The co-occurrence of OSA, smoking, and obstructive airway disease within the same individual amplifies respiratory disease burden and complicates both diagnosis and management, pointing to the need for integrated population-based assessments that capture these overlapping risk dimensions simultaneously.

Despite growing awareness of CRD burden at the national level, population-based epidemiological data from Romanian coastal communities remain scarce. The present cross-sectional study aimed to estimate the prevalence of diagnosed CRDs among 979 adults across five coastal localities in the Mangalia area. Secondary objectives were to characterise smoking patterns and novel tobacco use in relation to respiratory symptoms and airflow obstruction; to identify individuals at risk for sleep apnea and spirometric airflow obstruction, including those without a prior diagnosis; and to assess healthcare utilisation, follow-up patterns, and vaccination coverage within this population.

## 2. Materials and Methods

### 2.1. Study Design and Setting

This cross-sectional observational study was conducted over six days (11–16 August 2025) in five coastal localities within the Mangalia municipality, Constanța County, Romania: Neptun (*n* = 147), Olimp (*n* = 178), Saturn (*n* = 187), Mangalia (*n* = 257), and Jupiter (*n* = 210), comprising a total of 979 participants. No formal sample-size calculation was performed a priori, as recruitment relied on a non-probabilistic convenience strategy detailed in Section 2.2; the target enrolment was pragmatically set to approximate 1000 participants across the six-day campaign based on anticipated daily footfall at each site, consistent with comparable community screening initiatives. A cross-sectional community screening design was chosen specifically because it enables simultaneous, same-visit assessment of multiple modifiable respiratory risk domains (spirometry, smoking behaviour, sleep-apnea risk, vaccination status), allowing identification of co-occurring, previously unrecognised risk profiles–for example, undiagnosed obstruction combined with active smoking and low vaccination coverage–that would not be visible from any single specialty’s records, and that are consistent with the low preventive-care utilisation indicators observed directly in this cohort (13.9% recent spirometry uptake, 3.4% pneumococcal vaccination, 5% pulmonology follow-up). The different data domains were integrated at two complementary levels: descriptive analyses were used to characterise co-occurring clinical and behavioural profiles across respiratory symptoms, smoking exposure, spirometric findings, OSA-risk screening, vaccination status, and healthcare utilisation, while prespecified multivariable logistic regression models were used to identify independent predictors of the principal outcomes. Thus, the heterogeneous measures were interpreted as complementary components of a single community respiratory-risk profile rather than as a single composite outcome.

### 2.2. Participants and Recruitment

The study was conducted as a community-based respiratory screening campaign across five coastal localities in the Mangalia municipality. Participants were recruited through a combination of opportunistic street recruitment, public poster announcements, and a dedicated screening event, at outdoor and temporary screening stations established within each locality. Participation was voluntary and open to all adults aged ≥18 years present in the area during the campaign period, including permanent residents, seasonal workers, and tourists. No population-based random sampling frame was employed. Findings should therefore be interpreted as proportions observed among screening participants rather than as population-representative prevalence estimates; health-aware or symptomatic individuals may be overrepresented relative to the general coastal population. For brevity and consistency, the term “prevalence” is used throughout the manuscript to refer to the proportion of participants meeting the specified criteria within the screened study population; these estimates should not be interpreted as population-representative prevalence estimates because of the non-random, self-selected recruitment strategy. Residency/occupational status (resident, seasonal worker, or tourist) was not recorded as a structured questionnaire item; while informally reported by many participants during recruitment, these categories could not be systematically quantified and are not analysed further, representing a limitation discussed in Section 4. Inclusion required age ≥ 18 years and presence at a screening station during the campaign period; no other exclusion criteria were applied. Missing data were minimal for the principal variables: spirometry results were unavailable for 6/979 participants (0.6%), and item-level missingness for other key variables is reported alongside the corresponding results (see Table 1).

### 2.3. Data Collection

Data were collected using a structured, paper-based questionnaire comprising 22 domains, including demographic characteristics, CRD diagnoses, occupational exposures, allergic conditions, smoking history (including pack-years), use of novel tobacco products (electronic cigarettes, heated tobacco products, hookah, and nicotine pouches), respiratory symptoms (over the preceding six months), spirometry (FEV1 [L and % predicted], FVC [L and % predicted], and FEV1/FVC ratio), frequency and management of respiratory infections, activity limitation, healthcare utilisation (emergency visits and hospitalisations), treatment patterns, physician follow-up, timing of last pulmonology consultation, sleep characteristics, snoring and witnessed apnea, anthropometric measurements, shift work, vaccination status (influenza, pneumococcal, COVID-19), comorbidities (cardiovascular disease, diabetes mellitus, stroke), and CAT/ACT scores. To ensure consistency across localities, variables not uniformly collected across all sites were excluded from the pooled analysis. All chronic respiratory disease subtypes (chronic bronchitis, emphysema, COPD, and asthma) were captured solely as participant self-reports of a prior clinician diagnosis. No CT imaging or other chest imaging was performed as part of the present screening campaign, and no emphysema diagnosis was established or verified radiologically; likewise, CRD diagnoses were not verified against medical records or spirometric disease staging at the time of the screening encounter. Medication use, including inhaled corticosteroids and other inhaled respiratory therapies, was based on participant report and was not independently verified against prescriptions, dispensing records, or clinical medical records.

Spirometric measurements were performed using a Vitalograph Alpha (Vitalograph Ltd., Ennis, Ireland), equipped with the V-Core ultrasonic flow sensor, calibrated daily according to manufacturer specifications. All tests were conducted by resident physicians trained in spirometry technique, following the acceptability and reproducibility criteria of the American Thoracic Society/European Respiratory Society (ATS/ERS) 2019 guidelines. A minimum of three acceptable manoeuvres were obtained per participant, with the best FEV_1_ and FVC values used for analysis. Measurements were performed pre-bronchodilator; post-bronchodilator confirmation was not performed in this screening context, which may result in overestimation of airflow obstruction prevalence, particularly in older participants.

### 2.4. Derived Variables

Body mass index (BMI) was calculated as weight (kg) divided by height squared (m^2^), with obesity defined as BMI ≥ 30 kg/m^2^. Pack-years were calculated as (cigarettes per day ÷ 20) × years of smoking. For 9 participants with a valid value for only one component of this calculation (cigarettes per day or years smoked–the other recorded as a non-numeric response such as “occasional” or “cannot specify”), the missing component was imputed using the cohort median for that smoking-status group (current or former). Pack-years remained missing for participants lacking both components or with an unclassifiable smoking-status record. Airflow obstruction was defined as a pre-bronchodilator FEV1/FVC ratio < 0.70, in accordance with the fixed ratio (GOLD) criterion; spirometric data were available for 973 participants for obstruction classification; the corresponding multivariable model was fitted on 965 participants after listwise exclusion of missing covariate data, as detailed in Section 3.9.1. Sleep apnea risk was defined by the co-reporting of habitual snoring and witnessed apnoeic episodes, used as a two-item screening proxy for elevated OSA probability. This definition does not constitute a clinical diagnosis of OSA and should be interpreted as an inferred risk indicator; no apnea-hypopnea index (AHI), overnight oximetry, home sleep apnea testing (HSAT), or validated multi-item questionnaire (e.g., STOP-BANG, Berlin) was administered, and formal polysomnographic or polygraphic confirmation was not performed in this study. The presence of any CRD was defined as at least one of the following: chronic bronchitis (defined per standard criteria as a productive cough for ≥3 months in each of two consecutive years, distinguishing it from acute bronchitis; it may occur with or without airflow obstruction and is conventionally grouped within the COPD disease spectrum), emphysema, chronic obstructive pulmonary disease, or bronchial asthma. As an a posteriori sensitivity check for age-related misclassification inherent to the fixed 0.70 ratio, obstruction prevalence was examined across age strata: 10.3% (≤40 years), 13.2% (41–50 years), 19.0% (51–60 years), 34.1% (61–69 years), and 47.6% (≥70 years). This marked gradient is consistent with the known tendency of the fixed-ratio criterion to over-classify obstruction in older adults relative to a lower-limit-of-normal (LLN) approach; formal GLI-based LLN z-scores could not be computed retrospectively, as ethnicity—a required input for GLI-2012 reference equations—was not captured as a study variable, and this is noted as a limitation in Section 4. Findings in participants aged ≥65 years should accordingly be interpreted with additional caution.

### 2.5. Statistical Analysis

Data were analysed using JASP statistical software (version 0.97.0; https://jasp-stats.org). Categorical variables are presented as frequencies and percentages, while continuous variables are reported as mean ± standard deviation or median with interquartile range, depending on distribution as assessed by the Shapiro–Wilk test. Group comparisons were performed using the Chi-square or Fisher’s exact test for categorical variables, and the independent samples *t*-test or Mann–Whitney U test for continuous variables, as appropriate. Logistic regression analyses were conducted to identify predictors of chronic respiratory diseases and airflow obstruction. A two-sided *p*-value < 0.05 was considered statistically significant. Linearity in the logit for all continuous predictors (age, BMI, FEV1/FVC, height, weight, pack-years) was assessed using the Box-Tidwell test across all four multivariable models (Section 3.9); no significant departure from linearity was detected (all interaction *p* > 0.06). Multicollinearity was evaluated via variance inflation factors for all predictors in all four models, which ranged from 1.007 to 2.308, well below the threshold of concern (VIF > 5). Covariates for each multivariable model were selected a priori on the basis of clinical relevance and the prespecified study objectives rather than by univariate screening; the specific predictor set for each model is listed in the corresponding table. Descriptive comparisons (e.g., e.g., between localities and between diagnostic groups) used pairwise (available-case) analysis, whereas all four multivariable logistic regression models used listwise (complete-case) exclusion of participants with missing covariate data, as reported in the corresponding Results subsections. Formal calibration assessment (e.g., Hosmer–Lemeshow test) was not performed for any of the four models and is noted as a limitation in Section 4.

### 2.6. Ethics

The study was conducted in accordance with the Declaration of Helsinki, and approved by the Ethics Committee of the National Institute of Pneumology “Marius Nasta” (protocol code 14882, approved 29 July 2025). Written informed consent was obtained from all participants prior to enrolment. All data were anonymised and processed in accordance with the General Data Protection Regulation (GDPR) and Romanian Law 190/2018.

## 3. Results

As shown below, Table 2 provides a consolidated overview of the principal findings across the three core analytical domains of the study: demographic characteristics, respiratory disease burden, and key risk indicators. These results highlight the most clinically and epidemiologically relevant patterns, which are explored in detail in the subsequent subsections.

### 3.1. Study Population

**Figure 1:**
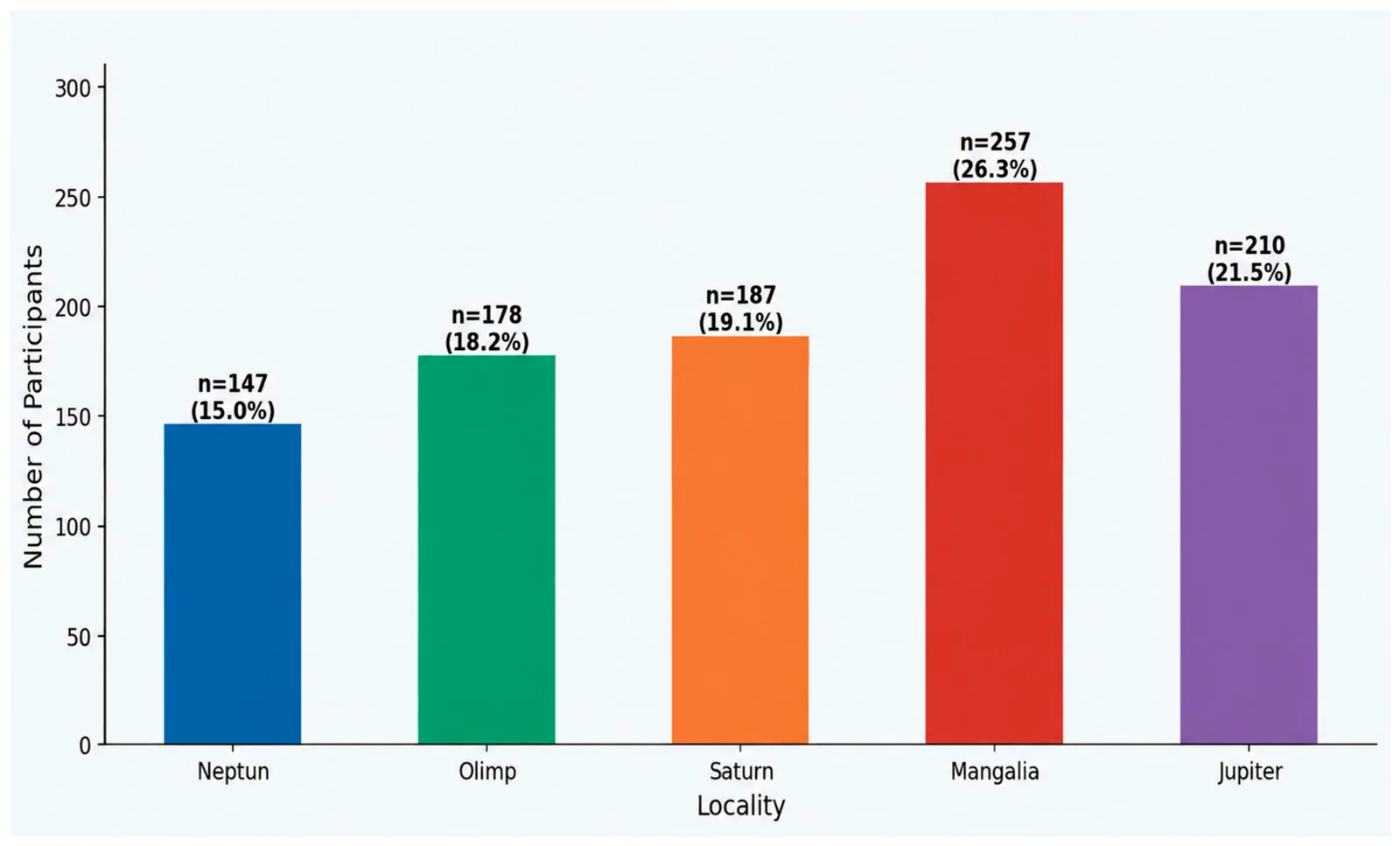
Participant distribution by locality. Mangalia contributed the largest share (*n* = 257, 26.3%).

A total of 979 adults were enrolled across five coastal localities: Neptun (*n* = 147, 15.0%), Olimp (*n* = 178, 18.2%), Saturn (*n* = 187, 19.1%), Mangalia (*n* = 257, 26.3%), and Jupiter (*n* = 210, 21.4%), as illustrated in Figure 1. The study population included 503 females (51.4%) and 474 males (48.4%), with a mean age of 47.3 ± 13.5 years (range 18–82). The largest age category was 41–60 years (*n* = 497, 50.8%).

Significant differences were observed across localities in both sex distribution (*p* = 0.028) and mean age (*p* < 0.001) (Table 3). Participants in Jupiter were the youngest on average (mean 43.1 years), whereas those in Neptun were the oldest (mean 50.8 years). Smoking prevalence also varied significantly between sites (*p* = 0.003), with the highest proportions of current smokers recorded in Mangalia (42%) and Olimp (40%).

The mean body mass index (BMI) across the cohort was 27.1 kg/m^2^, with no significant variation between localities (*p* = 0.149). In contrast, spirometry uptake within the preceding six months differed significantly (*p* = 0.005), with the lowest rate observed in Olimp (6%) and the highest in Mangalia and Jupiter (both 18%). Influenza vaccination coverage also showed marked variability (*p* < 0.001), ranging from 33% in Neptun to 12% in Jupiter.

### 3.2. Prevalence of Chronic Respiratory Diseases

**Figure 2:**
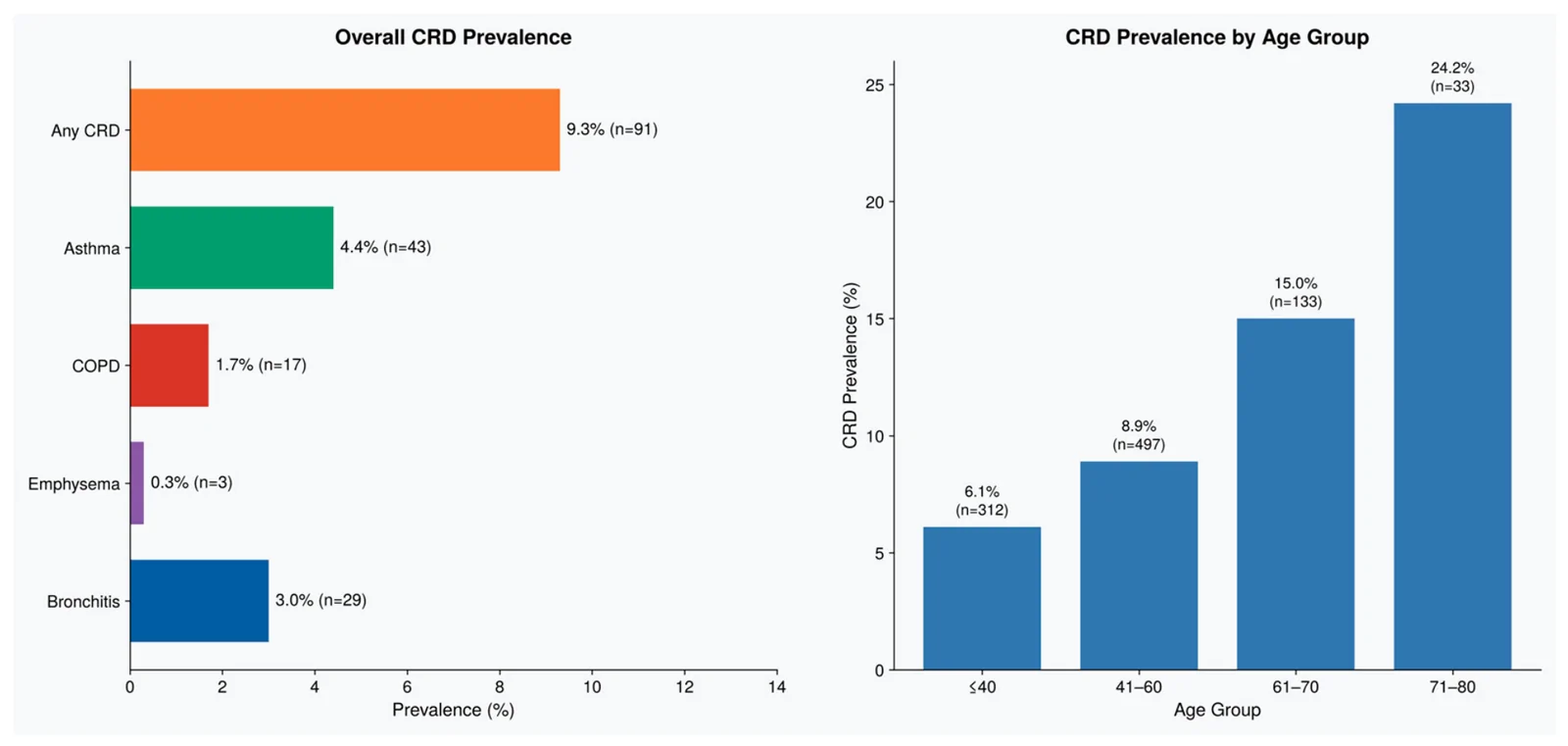
(**Left**): Overall CRD prevalence. (**Right**): CRD prevalence by age group–strong age gradient (6.1% to 24.2%).

As illustrated in Figure 2, the overall prevalence of chronic respiratory diseases (CRDs) was 9.3%, with a marked increase across age groups.

Among the 979 participants, 91 individuals (9.3%) reported at least one CRD diagnosis. Bronchial asthma was the most prevalent condition (*n* = 43, 4.4%), followed by chronic bronchitis (*n* = 29, 3.0%), chronic obstructive pulmonary disease (COPD) (*n* = 17, 1.7%), and emphysema (*n* = 3, 0.3%), as detailed in Table 4.

CRD prevalence did not differ significantly across localities (*p* = 0.265), suggesting a relatively uniform distribution of respiratory disease along the coastal region. In contrast, a pronounced age gradient was observed, with prevalence increasing from 6.1% in individuals under 40 years to 24.2% among those aged 71–80 years.

Sex-based differences were also evident. Female sex was associated with a significantly higher overall CRD prevalence compared to males (11.9% vs. 6.5%; absolute difference 5.4 percentage points; crude OR 1.935, 95% CI 1.230–3.045; *p* = 0.004), largely driven by the higher frequency of asthma among women (32 cases vs. 11 cases in men). In comparison, COPD and chronic bronchitis were more evenly distributed between sexes.

### 3.3. Airflow Obstruction and Spirometry

Spirometric data (FEV1/FVC ratio) were available for 973 of 979 participants, representing near-complete coverage of the study population. Overall, 174 individuals (17.9%) met the GOLD criterion for airflow obstruction (FEV1/FVC < 70%).

**Figure 3:**
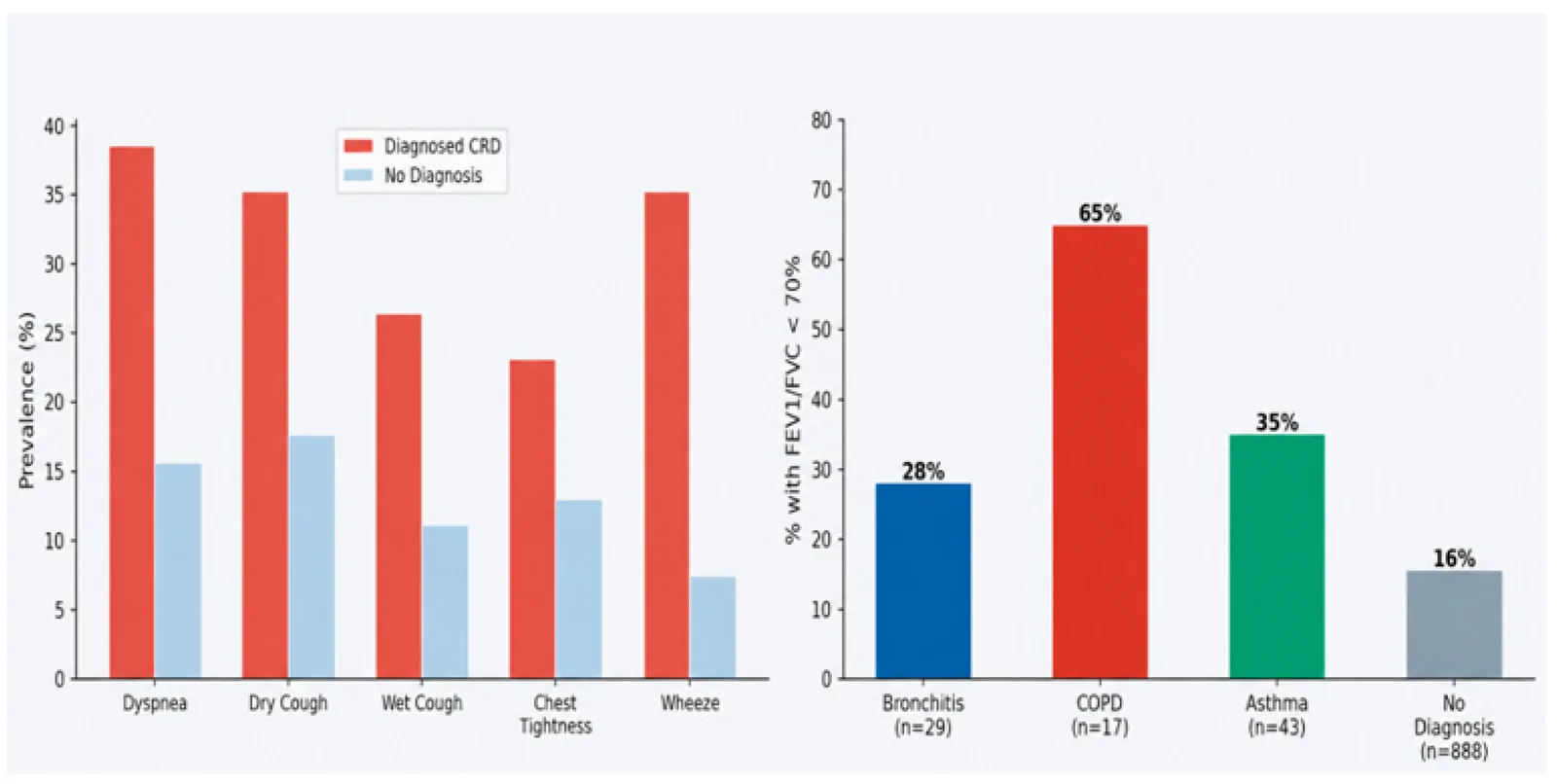
(**Left**): Symptom burden (diagnosed vs. undiagnosed). (**Right**): Airflow obstruction by diagnosis group.

As illustrated in Figure 3, airflow obstruction was most prevalent among participants with COPD (11/17, 65%), followed by those with asthma (15/43, 35%) and chronic bronchitis (8/29, 28%), supporting the internal consistency of reported diagnoses. However, a substantial proportion of obstruction was identified outside previously diagnosed cases. Among 888 participants without any recorded chronic respiratory disease, 139 (15.7%) demonstrated spirometric airflow limitation–representing previously unrecognised airflow limitation rather than confirmed chronic disease, as pre-bronchodilator spirometry alone is insufficient to establish a definitive COPD diagnosis without clinical confirmation.

This finding is particularly notable in the context of low spirometry utilisation, with only 136 of 979 participants (13.9%) reporting spirometric testing within the preceding six months. Together, these results highlight a significant diagnostic gap in routine respiratory assessment.

Mean FEV1/FVC ratio was significantly lower in participants with diagnosed CRDs compared to those without (71.9 ± 11.7% vs. 76.8 ± 8.8%, *p* < 0.001), further supporting the clinical relevance of the observed obstruction patterns.

### 3.4. Smoking and Novel Tobacco Use

Smoking exposure was highly prevalent within the cohort: 35.2% of participants were current smokers, 17.9% were former smokers, and 53.1% reported a history of smoking.

**Figure 4:**
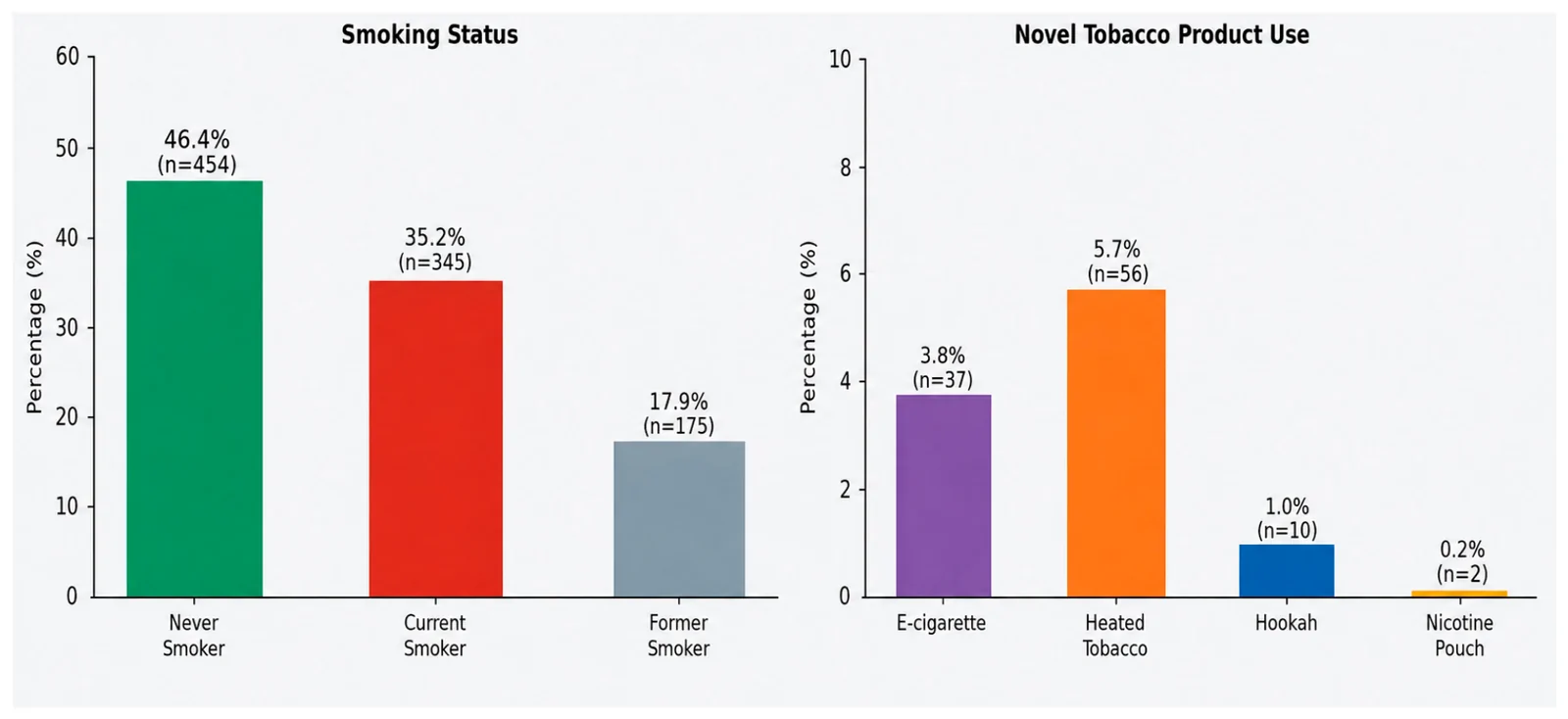
(**Left**): Smoking status. (**Right**): Novel tobacco product use—heated tobacco most common (5.7%).

As shown in Table 5 and Figure 4, smoking status did not differ significantly between participants with and without diagnosed chronic respiratory diseases (all *p* > 0.5). Similarly, the use of novel tobacco products was not associated with CRD diagnosis (all Fisher’s exact *p* > 0.7). These findings likely reflect both the relatively small size of the diagnosed CRD group (*n* = 91) and the widespread distribution of tobacco exposure across the study population.

Notably, participants with spirometric airflow obstruction exhibited the highest prevalence of current smoking (41%), suggesting a potential contribution of smoking to airflow limitation even in the absence of a formal diagnosis.

Among novel tobacco products, heated tobacco devices (e.g., IQOS) were the most commonly used (5.7%), followed by e-cigarettes (3.8%), hookah (1.0%), and nicotine pouches (0.2%), with similar usage patterns observed across diagnostic groups.

### 3.5. Sleep Apnea Risk–Inferred Finding

Sleep apnea risk was assessed using a two-item symptom-based screening proxy derived from self-reported habitual snoring and witnessed apneic episodes. This approach serves as an inferred risk indicator and does not constitute a clinical OSA diagnosis; formal polysomnographic or polygraphic confirmation was not performed. While validated multi-item tools such as STOP-BANG offer greater sensitivity and specificity, the two-item proxy used here captures the two strongest individual STOP-BANG predictors (snoring and observed apneas) and is consistent with screening frameworks used in primary care settings. Results should therefore be interpreted as an estimate of elevated OSA probability within this population.

**Figure 5:**
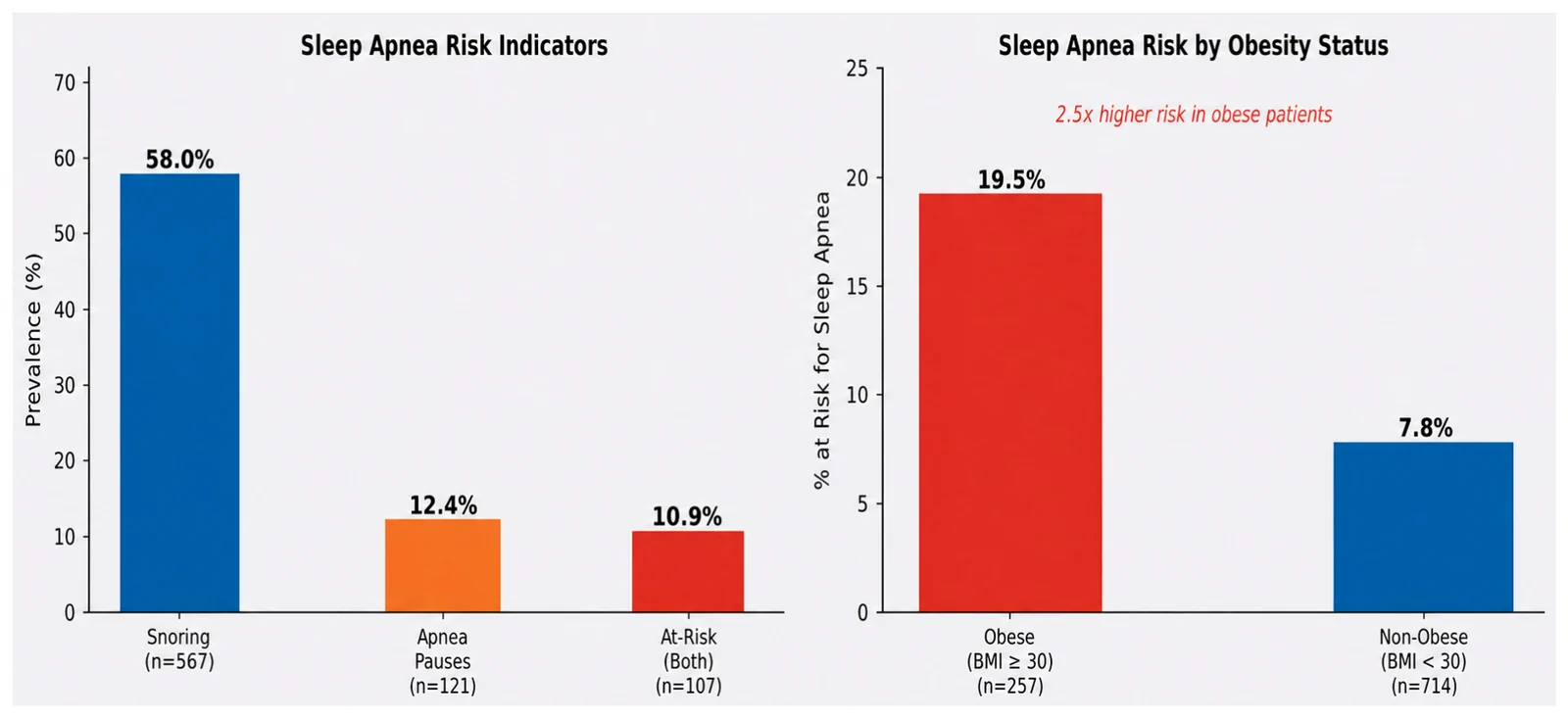
(**Left**): Apnea risk indicators. (**Right**): Screening-defined OSA-risk positivity is higher among obese than non-obese participants (19.5% vs. 7.9%; OR 2.82, 95% CI 1.87–4.25; *p* < 0.001).

Snoring was reported by 567 participants (57.9%), while witnessed breathing pauses were reported by 121 (12.4%). The combination of both criteria, used as a proxy for elevated obstructive sleep apnea (OSA) risk, was identified in 107 participants (10.9%), as illustrated in Figure 5.

A strong association with obesity was observed. Among individuals with a body mass index (BMI) ≥ 30 kg/m^2^, 50 of 257 (19.5%) met the at-risk criteria, compared to 57 of 722 (7.9%) among non-obese participants, corresponding to an OR of 2.82 (95% CI 1.87–4.25) for screening-defined OSA-risk positivity (*p* < 0.001).

Additional sleep-related risk indicators were also common. Short sleep duration (3–4 h per night) was reported by 184 participants (18.8%), and 125 (12.8%) reported insomnia. The mean BMI across the cohort was 27.1 kg/m^2^, with 26.3% of participants classified as obese.

Sex-stratified analysis of sleep apnea risk indicators revealed a marked male predominance. Habitual snoring was reported by 321 of 474 males (67.7%) compared with 246 of 503 females (48.9%), while witnessed apnoeic episodes were more frequent in males (71/474, 15.0%) than in females (50/503, 9.9%). These sex differences are consistent with established epidemiological patterns and likely reflect both anatomical and hormonal factors contributing to upper airway collapsibility. When stratified by obesity and co-occurring sleep modifications (insomnia or short sleep duration of 3–4 h/night), snoring and witnessed apneas were most prevalent in the obese subgroup with concurrent sleep modifications (*n* = 70): 80.0% reported snoring and 31.4% reported witnessed apnoeic episodes. Obese individuals without sleep modifications (*n* = 187) showed similarly high snoring rates (77.0%) but fewer witnessed apneas (17.1%), while non-obese participants (*n* = 722) had substantially lower rates of both indicators (50.8% and 9.3%, respectively). These findings highlight the additive burden of obesity combined with sleep disturbance on OSA risk indicators in this population, as summarized in Table 6.

Overall, these findings suggest a substantial burden of unrecognised sleep apnea risk within this population, with obesity showing the strongest association with OSA risk in this cohort.

### 3.6. Respiratory Symptoms (Past 6 Months)

Respiratory symptoms within the preceding six months were frequently reported across the cohort. Dry cough was the most common symptom (188/979, 19.2%), followed by dyspnoea (174/979, 17.8%), chest tightness (136/979, 13.9%), productive cough (124/979, 12.7%), and wheezing (99/979, 10.1%).

Importantly, a substantial symptom burden was also observed among participants without a diagnosed chronic respiratory disease. In this group, 15.7–17.6% reported dry cough or dyspnoea, while 7.5–13.0% reported wheezing or chest tightness.

The presence of respiratory symptoms in ostensibly undiagnosed individuals is consistent with the observed prevalence of spirometric airflow obstruction in this subgroup and further supports the likelihood of underdiagnosis within the population. These findings reinforce the need for earlier identification strategies, including targeted symptom-based screening and broader use of spirometry.

### 3.7. Healthcare Utilisation and Vaccination

**Figure 6:**
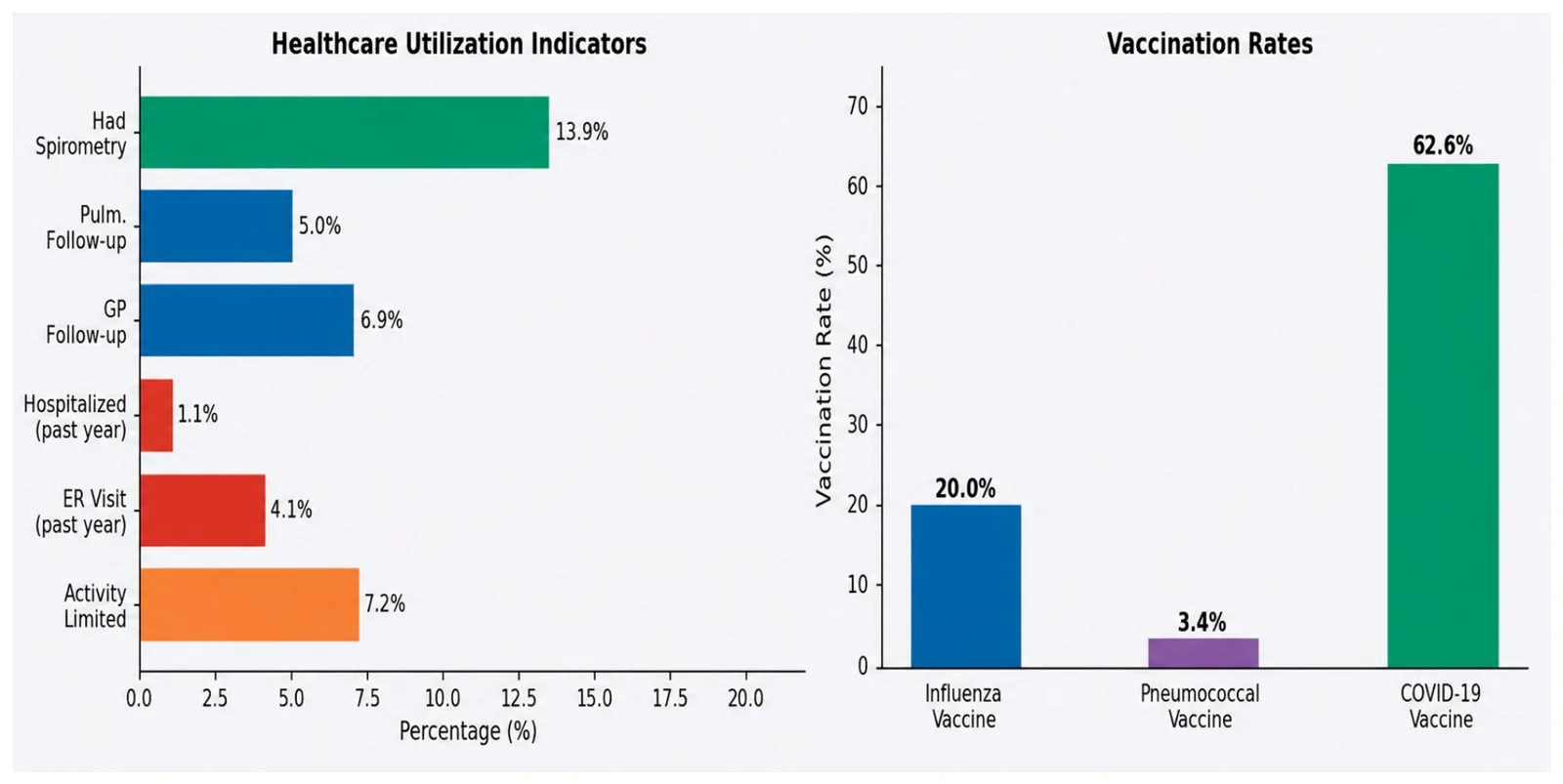
(**Left**): Utilisation indicators. (**Right**): Vaccination—COVID 62.5%, influenza 20.0%, pneumococcal only 3.4%.

Healthcare utilisation and vaccination patterns are summarised in Table 7 and illustrated in Figure 6.

Participants with a diagnosed chronic respiratory disease demonstrated substantially higher engagement with healthcare services. Spirometry within the preceding six months was reported by 44% of CRD-positive individuals compared to 11% of those without a diagnosis (*p* < 0.001). Similarly, emergency department visits were more frequent in the CRD group (10% vs. 3%, *p* = 0.008), as were hospitalisations (5% vs. 1%, *p* = 0.002).

A further comparison between participants with previously known CRD (*n* = 91) and those with airflow obstruction newly identified at screening (*n* = 139) revealed even more pronounced disparities, particularly in specialist follow-up (pulmonologist follow-up: 45.1% vs. 1.4%) and activity limitation (38.5% vs. 4.3%), as detailed in Table 8.

These healthcare utilisation indicators were analysed descriptively in the present cross-sectional study to characterise differences in prior healthcare engagement and to contextualise the observed diagnostic gap; they were not included as predictors in the primary multivariable models, which were designed to examine CRD diagnosis, spirometric airflow obstruction, OSA-risk screening positivity, and diagnosis within the obstructed subgroup.

Marked differences were also observed in follow-up care. Pulmonologist follow-up was reported by 45% of participants with CRDs, compared to only 1% of those without a diagnosis (*p* < 0.001), highlighting a substantial disparity in access to specialist care. General practitioner follow-up showed a similar pattern (29% vs. 5%, *p* < 0.001). In addition, activity limitation attributed to respiratory disease was significantly more common among CRD-positive individuals (38% vs. 4%, *p* < 0.001).

Vaccination coverage showed contrasting patterns. COVID-19 vaccination uptake was relatively high (62.5%) and did not differ significantly between groups (*p* = 0.587). In contrast, influenza vaccination coverage was low (20.0%) and similarly showed no difference by diagnosis status (*p* = 0.724). Pneumococcal vaccination uptake was critically low at 3.4% overall, with no significant variation between CRD-positive and undiagnosed participants (5% vs. 3%, *p* = 0.223), remaining far below recommended targets for individuals with chronic respiratory conditions. When stratified by age, pneumococcal uptake increased from 1.6% in participants aged ≤40 years to 3.0% among those aged 41–60 years and 10.8% among those aged ≥65 years. This age-banded comparison targets the clinically relevant ≥ 65-year eligibility threshold rather than providing an exhaustive age-stratified breakdown; participants aged 61–64 are not separately reported. Restricting the analysis to participants meeting standard clinical eligibility criteria for pneumococcal vaccination (age ≥ 65 years or a pre-existing CRD diagnosis, *n* = 183), coverage remained low at 8.2%, compared with 2.3% among non-eligible participants–confirming that the shortfall persists even within the clinically indicated subgroup and is not merely an artefact of a predominantly younger, non-eligible sample.

Overall, these findings highlight substantial gaps in preventive care and early diagnostic engagement, particularly among individuals without a formal respiratory diagnosis.

### 3.8. Allergies and Comorbidities

Allergic conditions were notably more prevalent among participants with chronic respiratory diseases compared to the overall cohort. House dust allergy was the most common allergy overall (9.2%), followed by pollen allergy (7.9%) and other allergies (16.1%). Among CRD-positive individuals, however, the prevalence was substantially higher, with house dust allergy reported by 23%, pollen allergy by 22%, and mould allergy by 15%, indicating a strong association between atopic burden and respiratory disease. When stratified by self-reported diagnosis, allergy burden was highest among participants with self-reported asthma (67% any allergy), intermediate in chronic bronchitis (48%), and absent in the three emphysema cases, as detailed in Table 9.

Cardiovascular comorbidity was common in the cohort (19.7%), although its association with CRD diagnosis did not reach statistical significance (*p* = 0.070). Diabetes mellitus was present in 6.1% of participants and showed no association with CRD status (*p* = 1.000). In contrast, a history of stroke was significantly more frequent among CRD-positive individuals (5.5% vs. 1.6%, *p* = 0.025), potentially reflecting shared vascular risk factors or the systemic consequences of chronic respiratory impairment.

Lung function analysis further supported the clinical findings. The mean FEV_1_/FVC ratio was significantly lower in participants with CRDs compared to those without a diagnosis (71.9 ± 11.7% vs. 76.8 ± 8.8%, *p* < 0.001), consistent with the expected physiological differences associated with chronic respiratory disease.

#### Sensitivity Analysis: Multiple Comparisons Adjustment

Given the number of univariate comparisons reported in the descriptive tables (21 tests across Table 3′s locality comparisons and Table 7’s healthcare-utilisation/vaccination comparisons by diagnosis status), a Benjamini-Hochberg false discovery rate (FDR) correction was applied post hoc to assess robustness to multiple testing (Table 10). Of the 11 comparisons significant at the unadjusted *p* < 0.05 threshold, 10 remained significant after FDR correction; only the between-locality difference in sex distribution (raw *p* = 0.028, FDR-adjusted *p* = 0.053) fell below the corrected significance threshold. This indicates that the principal descriptive findings—including smoking prevalence, spirometry uptake, and healthcare utilisation differences—are robust to correction for multiple comparisons, with the exception of the locality-level sex distribution, which should be interpreted with caution.

### 3.9. Logistic Regression Analysis

Four multivariable logistic regression models were constructed to identify independent predictors of the principal clinical outcomes: predictors of CRD diagnosis (Table 11), predictors of spirometric airflow obstruction (Table 12), predictors of OSA screening risk (Table 13), and, within the obstructed subgroup, predictors of having received a prior diagnosis (Table 14). Given documented between-locality heterogeneity in several model covariates (Table 3), each of these four models was additionally refitted with locality as a sensitivity check (Section 3.9.4).

To identify independent predictors of chronic respiratory disease diagnosis, a binary logistic regression model was constructed with CRD status (yes/no) as the dependent variable and age, FEV_1_/FVC ratio, height, weight, and sex as covariates (*n* = 969; 10 participants excluded due to missing data in one or more covariates). The results are presented in Table 11.

After adjustment for all variables, increasing age was significantly associated with higher odds of CRD diagnosis (OR 1.022, 95% CI 1.001–1.041, *p* = 0.028). In parallel, a lower FEV_1_/FVC ratio emerged as the strongest predictor (OR 0.958, 95% CI 0.936–0.981, *p* < 0.001), consistent with an independent association between reduced airflow and reported CRD diagnosis in this model. Moreover, female sex was independently associated with more than a twofold increase in the odds of CRD (OR 2.224, 95% CI 1.158–4.270, *p* = 0.016).

In contrast, neither height nor weight showed a significant association after adjustment (*p* = 0.580 and *p* = 0.163, respectively). Overall, the model demonstrated modest explanatory power (Nagelkerke R^2^ = 0.079), consistent with the multifactorial nature of CRDs in a community-based population.

#### 3.9.1. Predictors of Spirometric Airflow Obstruction

A binary logistic regression model was fitted with spirometric airflow obstruction (FEV_1_/FVC < 70%) as the dependent variable and nine clinical and demographic predictors as covariates (*n* = 965; participants with missing spirometry or covariate data were excluded). The results are presented in Table 12.

After adjustment for all covariates, older age (OR 1.054, 95% CI 1.038–1.071, *p* < 0.001) and greater cumulative tobacco exposure, measured in pack-years (OR 1.013, 95% CI 1.002–1.024, *p* = 0.025), emerged as the only independent predictors of airflow obstruction. Sex, BMI, current smoking status, novel tobacco use, pneumococcal vaccination status, OSA risk, and occupational exposure were not significantly associated with airflow obstruction after adjustment.

These findings suggest that airflow obstruction in this population was independently associated with age and cumulative tobacco exposure rather than current smoking behavior, consistent with the progressive and time-dependent nature of obstructive airway disease. The model demonstrated moderate explanatory power (Nagelkerke R^2^ = 0.131). Because pack-years remained independently associated with obstruction after adjustment, whereas BMI and OSA risk did not show statistically significant independent associations, the findings support cumulative tobacco exposure as the principal independent smoking-related predictor in this model; they provide no evidence of an independent contribution of obesity or OSA risk to airflow obstruction.

Consistent with this pattern of greater cumulative tobacco exposure, obstruction prevalence among current smokers increased monotonically with cumulative exposure: 16.9% at 1–10 pack-years, 20.2% at 11–20 pack-years, and 25.8% at >20 pack-years, although the simple unadjusted correlation between pack-years and FEV1/FVC ratio among current smokers with complete pack-year data (*n* = 327) was weak (r = −0.042, *p* = 0.450), underscoring the importance of the age-adjusted multivariable estimate over a crude bivariate association. After adjustment, cumulative tobacco exposure (pack-years) remained an independent predictor in the fully adjusted model (Table 12; OR 1.013 per pack-year, *p* = 0.025), whereas the binary current-smoker variable did not reach significance after adjustment (OR 1.343, *p* = 0.175), indicating that cumulative dose, rather than current smoking status per se, is the more robust independent predictor.

Because the present study was designed primarily to characterise screening-detected airflow obstruction rather than to define a composite clinical severity phenotype, we did not construct an additional composite outcome combining respiratory symptoms, smoking exposure, BMI, and OSA risk. Instead, these variables were evaluated simultaneously as covariates in the multivariable model of airflow obstruction (Table 12), allowing assessment of their independent associations with spirometric obstruction. In this model, age and cumulative tobacco exposure remained independently associated with obstruction, whereas BMI and OSA risk did not reach statistical significance after adjustment. The symptom-defined subgroups presented in Section 3.10.1 are therefore intended as descriptive clinical phenotypes rather than as a separately modelled severity outcome. This approach avoids introducing a post hoc composite endpoint that was not prespecified and should be interpreted as hypothesis-generating.

#### 3.9.2. Predictors of Sleep Apnea Screening Risk

Elevated OSA screening risk (defined as co-reporting of habitual snoring and witnessed apneic episodes) was modeled as the dependent variable in a multivariable logistic regression analysis (*n* = 965, participants with missing covariate data, including airflow obstruction status, were excluded). The results are presented in Table 13.

BMI emerged as the strongest independent predictor of OSA screening risk (OR 1.115 per unit increase, 95% CI 1.072–1.160, *p* < 0.001), supporting excess adiposity as the principal modifiable risk factor in this population. Current smoking was independently associated with nearly a twofold increase in the odds of meeting OSA risk criteria compared to non-smokers (OR 1.958, 95% CI 1.259–3.045, *p* = 0.003). Older age was also a significant predictor (OR 1.023, 95% CI 1.005–1.041, *p* = 0.014).

Female sex was independently associated with a lower likelihood of OSA risk (OR 0.608, 95% CI 0.392–0.943, *p* = 0.026), in line with the well-established male predominance of obstructive sleep apnea. In contrast, CRD diagnosis and airflow obstruction were not independently associated with OSA risk after adjustment.

The model demonstrated moderate explanatory power (Nagelkerke R^2^ = 0.120).

#### 3.9.3. Predictors of CRD Diagnosis Among Participants with Airflow Obstruction

Of the 174 participants with spirometric airflow obstruction (FEV_1_/FVC < 70%), 35 (20.1%) had a prior CRD diagnosis, while 139 (79.9%) were previously undiagnosed. Predictors of having received a diagnosis versus remaining undiagnosed were evaluated using a multivariable logistic regression model within this obstructed subgroup (*n* = 172 after exclusions for missing covariate data). Given the limited number of events (35 diagnosed cases), the model was restricted a priori to three clinically prioritised predictors (age, sex, BMI) to maintain an adequate events-per-variable ratio (approximately 11.7) and reduce overfitting risk; pack-years, current smoking, OSA risk, and pneumococcal vaccination status were not retained in this parsimonious model and are instead examined descriptively in Section 3.10. The results of the reduced model are presented in Table 14.

Older age (OR 1.055, 95% CI 1.018–1.094, *p* = 0.004) and higher BMI (OR 1.100, 95% CI 1.009–1.198, *p* = 0.030) were independently associated with a greater likelihood of having received a prior CRD diagnosis within the obstructed subgroup. Female sex showed a positive but non-significant association (OR 2.184, 95% CI 0.971–4.914, *p* = 0.059).

These findings indicate that, among individuals with screening-detected pre-bronchodilator airflow obstruction, those who remain undiagnosed tend to be younger and have lower BMI, highlighting a potential gap in early case detection among younger, leaner individuals presenting to primary care. Given the exploratory nature of this reduced model and the modest number of events, these associations should be interpreted as hypothesis-generating rather than confirmatory.

#### 3.9.4. Sensitivity Analysis: Locality-Adjusted Models

Table 3 demonstrated significant between-locality differences in age (*p* < 0.001), sex distribution (*p* = 0.028), and current-smoking prevalence (*p* = 0.003)—each a covariate in the four multivariable models above (Table 11, Table 12, Table 13 and Table 14), none of which included locality as a covariate, stratifying factor, or random effect. To assess whether these between-site differences could be confounding the reported associations, we refitted each of the four models with locality added as a fixed categorical covariate (five levels: Neptun, Olimp, Saturn, Mangalia, Jupiter; reference category: Mangalia, the largest site), and additionally fitted random-intercept (mixed-effects) logistic models for airflow obstruction and OSA-risk—from shared local environmental or occupational exposures (Table 15).

For airflow obstruction (Table 12), adding locality did not materially change the principal associations: age (OR 1.052 vs. 1.054 without locality, both *p* < 0.001) and pack-years (OR 1.012 vs. 1.013, *p* = 0.057 vs. 0.025) retained essentially the same magnitude and direction, and the locality block itself was not statistically significant (*p* = 0.457). A random-intercept mixed-effects model corroborated this, with a modest estimated locality-level variance (SD = 0.22, 95% CI 0.11–0.44).

For OSA-risk (Table 13), locality as a block was statistically significant (χ^2^ = 10.18, df = 4, *p* = 0.038), driven specifically by higher OSA-risk in Olimp relative to Mangalia (OR 1.97, *p* = 0.028)—plausibly reflecting site-level differences in occupational shift work, local environmental exposures, or other unmeasured local factors. However, the principal individual-level predictors were not meaningfully altered by this adjustment: BMI (OR 1.127 vs. 1.115, both *p* < 0.001), current smoking (OR 1.913 vs. 1.958, both *p* < 0.01), age (OR 1.020 vs. 1.023, both *p* < 0.05), and female sex (OR 0.594 vs. 0.608, both *p* < 0.05) remained independently associated with OSA-risk in the same direction and similar magnitude. A random-intercept model showed a larger locality-level variance component for this outcome (SD = 0.37, 95% CI 0.19–0.72) than for obstruction, consistent with locality carrying independent explanatory value specific to OSA-risk.

For CRD diagnosis (Table 11) and diagnosis within the obstructed subgroup (Table 14), locality was not a statistically significant block predictor (*p* = 0.486 and *p* = 0.274, respectively), and the principal covariate estimates were essentially unchanged after adjustment (Table 15).

Taken together, these sensitivity analyses indicate that the between-locality demographic and smoking-prevalence heterogeneity documented in Table 3 does not materially confound the principal associations reported in Table 11, Table 12, Table 13 and Table 14. The one partial exception is OSA-risk, for which locality carries additional, statistically significant explanatory value beyond the modelled individual-level predictors.

### 3.10. Clinical Risk Profiles–Cross-Cutting Subgroup Analysis

To achieve a more clinically refined characterization of the study population, participants were stratified into risk profiles across three intersecting dimensions: (1) the coexistence of respiratory symptoms and screening-detected pre-bronchodilator airflow obstruction; (2) smoking status in relation to the presence of obstruction; and (3) the risk of obstructive sleep apnea in relation to obstruction. Additionally, all subgroups were further stratified by age (18–30, 31–45, 46–60, >60 years) and body mass index categories.

#### 3.10.1. Classification by Symptomatology and Bronchial Obstruction

**Figure 7:**
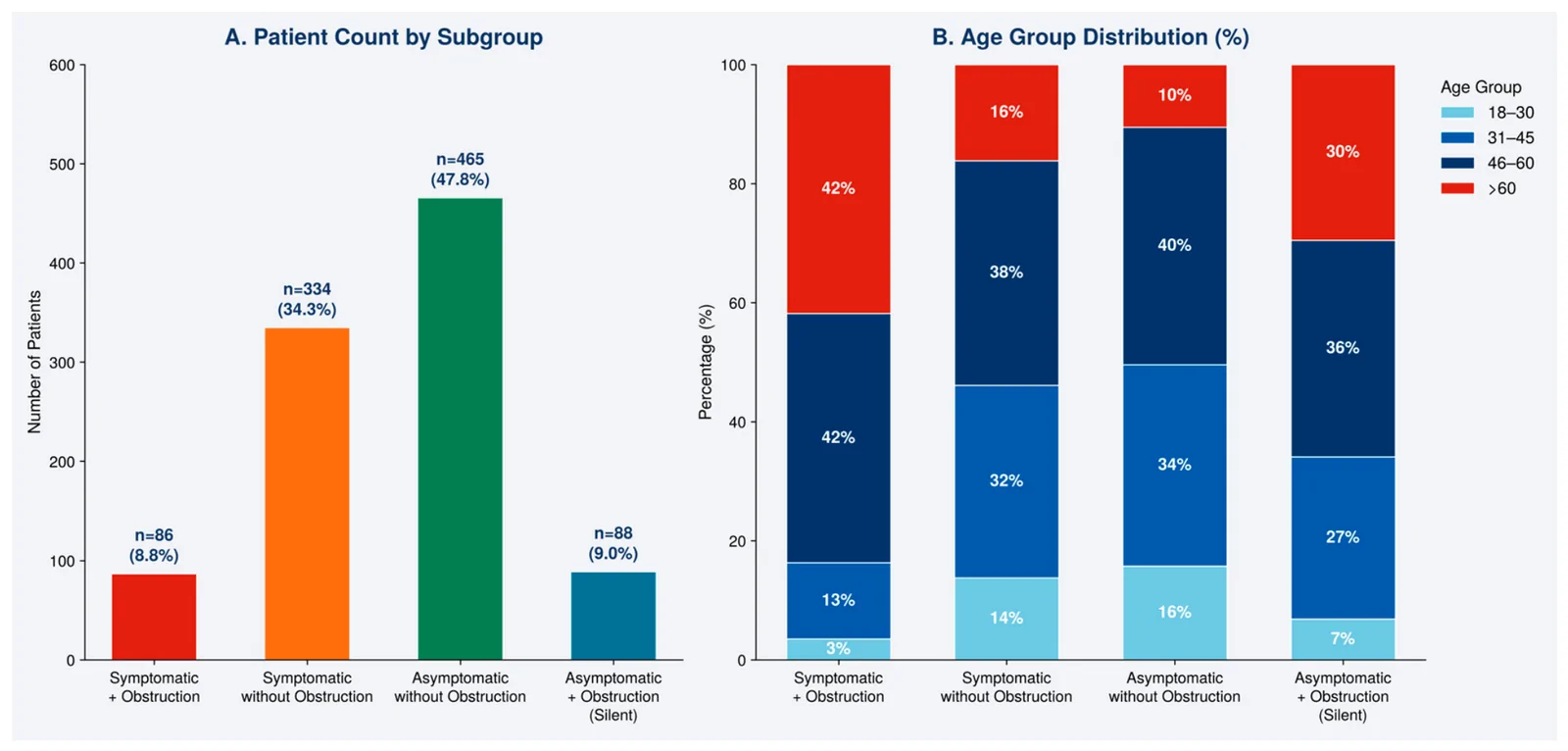
Distribution of patients according to symptomatology and obstruction status. Panel (**A**) illustrates the absolute number of patients in each subgroup, while Panel (**B**) depicts the proportional distribution of age categories within each subgroup. Stacked bar charts represent the four predefined age groups: 18–30, 31–45, 46–60, and >60 years. Percentages in panel B are rounded to whole numbers so that each bar totals 100%; exact values are reported in Section 3.10.4.

Participants were categorized into four clinically relevant subgroups based on the presence or absence of respiratory symptoms and spirometrically defined airflow obstruction (FEV_1_/FVC < 70%), as presented in Figure 7 and detailed in Table 16.

The subgroup comprising symptomatic individuals with screening-detected pre-bronchodilator airflow obstruction (*n* = 86, 8.8%) reflects the highest clinical burden within the cohort. This group exhibited the highest mean age (56.7 ± 12.9 years), the lowest mean FEV_1_/FVC ratio (61.1 ± 10.4%), and the greatest prevalence of chronic respiratory disease diagnoses (32.6%). This prevalence is nearly eightfold higher compared to the asymptomatic, non-obstructive subgroup (4.3%). Furthermore, a substantial majority of patients in this category (83.7%) were aged 46 years or older, emphasizing the significant contribution of age to the coexistence of symptoms and airflow limitation.

In contrast, the asymptomatic subgroup with screening-detected pre-bronchodilator airflow obstruction (*n* = 88, 9.0%)–representing individuals with clinically silent airflow limitation–holds particular clinical relevance. Despite the absence of reported respiratory symptoms, these patients demonstrated a reduced mean FEV_1_/FVC ratio (64.2 ± 5.3%) and a CRD prevalence of 8.0%. These findings highlight the potential for underdiagnosis and underscore the value of spirometric screening, even in apparently asymptomatic individuals.

#### 3.10.2. Classification by Smoking Status and Bronchial Obstruction

**Figure 8:**
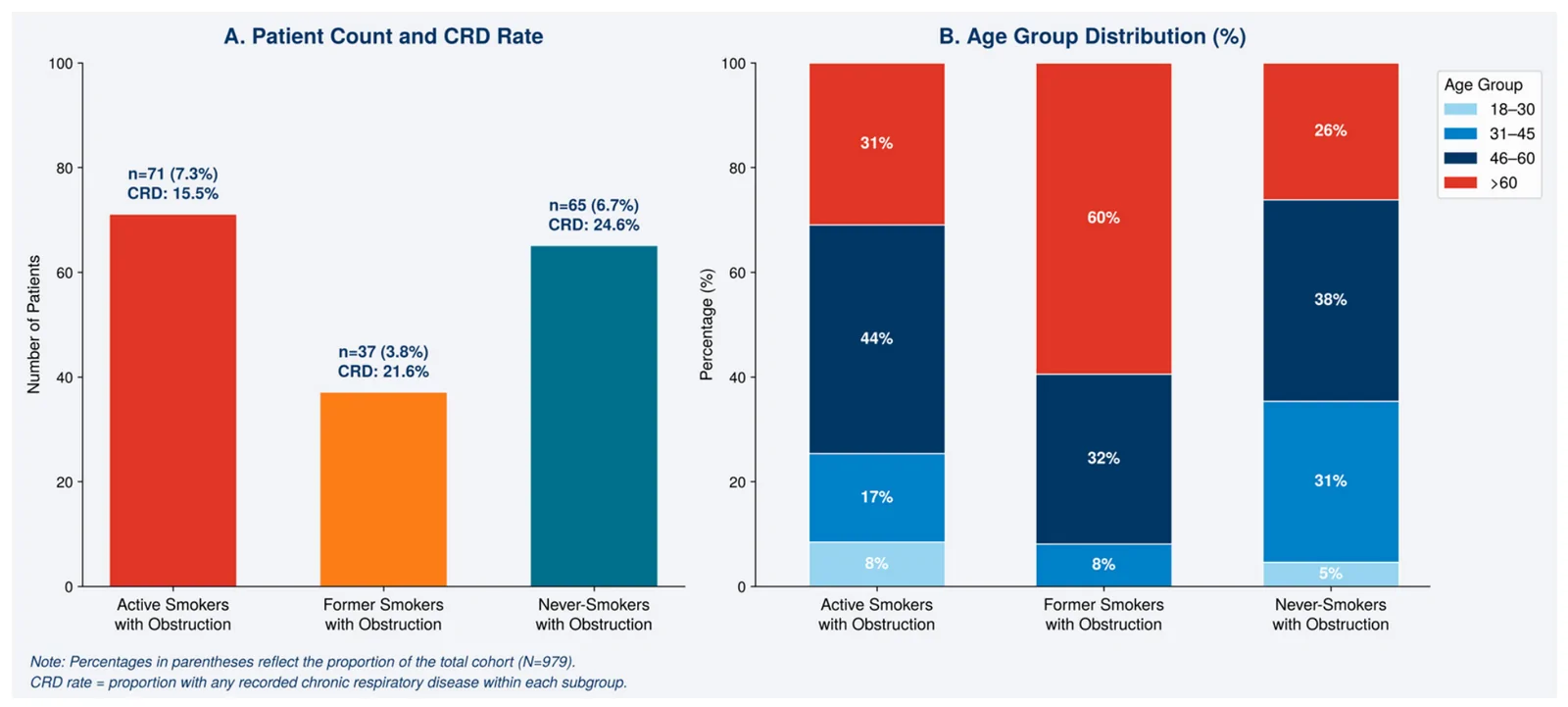
Distribution of patients with screening-detected pre-bronchodilator airflow obstruction according to smoking status. Panel (**A**) presents the number of patients and the proportion with a chronic respiratory disease diagnosis within each subgroup, while Panel (**B**) illustrates the age distribution across subgroups. Percentages in panel B are rounded to whole numbers so that each bar totals 100%; exact values are reported in Section 3.10.4.

Among participants with screening-detected pre-bronchodilator airflow obstruction (FEV_1_/FVC < 70%), three subgroups were defined according to smoking history: current smokers, former smokers, and never-smokers. The distribution of these groups is illustrated in Figure 8 and detailed in Table 17.

A notable finding is the gradient observed in CRD diagnosis rates across smoking categories. Never-smokers with screening-detected pre-bronchodilator airflow obstruction exhibited the highest prevalence of CRD (24.6%), followed by former smokers (21.6%) and current smokers (15.5%). This pattern is examined in detail below, including disaggregation of CRD subtype by smoking status both across the whole cohort and within this obstructed subgroup (Table 18 and Table 19), and the sex distribution across smoking-status groups (Table 20), given that female sex is an independent predictor of CRD diagnosis in this cohort (Table 11).

Given the marked difference in sex composition across smoking-status groups (Table 20) and the known independent association between female sex and CRD diagnosis (Table 11), we fitted a logistic regression model of asthma diagnosis (whole cohort, *n* = 974) with smoking status and sex as covariates. Female sex remained independently associated with asthma after adjustment for smoking status (adjusted OR 2.62, 95% CI 1.29–5.34, *p* = 0.008–a distinct, asthma-specific estimate from the overall-CRD model in Table 11, OR 2.224), while the smoking-status–asthma association remained non-significant and was not strengthened by adjustment for sex (current-vs-never-smoker OR 0.61 unadjusted, *p* = 0.285, versus 0.63 sex-adjusted, *p* = 0.212; former-vs-never-smoker OR 0.57 unadjusted, *p* = 0.122, versus 0.77 sex-adjusted, *p* = 0.571).

Returning to the descriptive characteristics of the obstructed subgroups in Table 17, former smokers with screening-detected pre-bronchodilator airflow obstruction were characterized by a higher mean age (61.5 ± 9.7 years) and a marked male predominance (70.3%), consistent with the classical epidemiological profile of chronic obstructive pulmonary disease. Age distribution further supports this pattern: 59.5% of former smokers were aged >60 years, compared to 31.0% of current smokers and 26.2% of never-smokers, indicating a cumulative exposure effect and age-related disease manifestation.

With regard to obesity and OSA-risk burden among current smokers with obstruction, Table 17 indicates a mixed picture. Mean BMI among current smokers with obstruction (27.1 kg/m^2^) was identical to that of never-smokers with obstruction and lower than that of former smokers with obstruction (28.1 kg/m^2^), indicating that current smoking and elevated BMI did not co-cluster within the obstructed subgroup. OSA-risk prevalence followed a different pattern: 23.9% of current smokers with obstruction met screening criteria for elevated OSA risk, compared with 13.5% of former smokers and 9.2% of never-smokers with obstruction (χ^2^ test, *p* = 0.059), a difference of borderline statistical significance given the modest subgroup sizes. Current smokers with obstruction were therefore not distinguished by higher BMI, but showed a numerically higher, though not definitively confirmed, burden of OSA-risk relative to the other obstructed smoking subgroups.

#### 3.10.3. Classification by OSA Risk and Bronchial Obstruction

**Figure 9:**
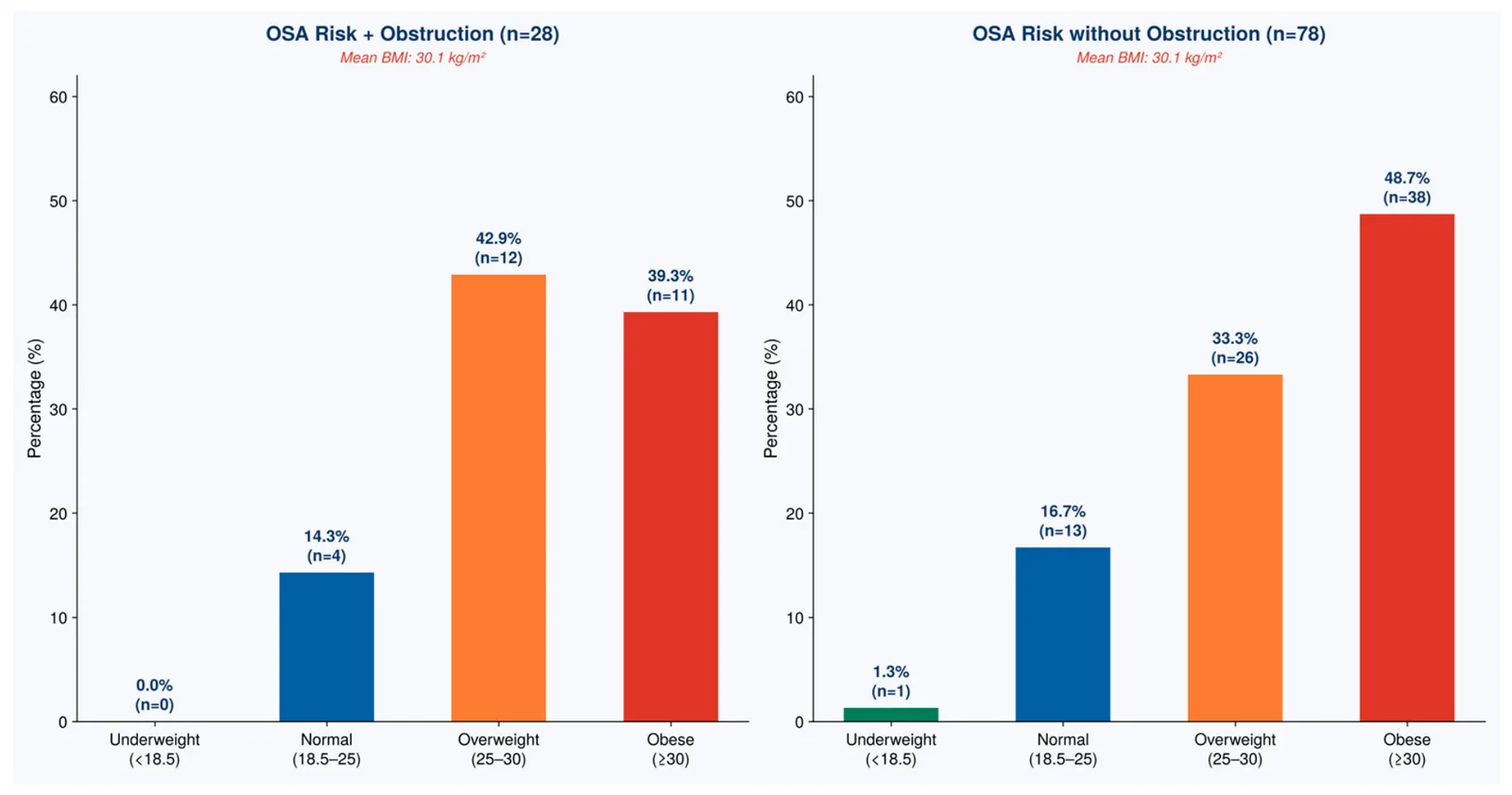
BMI distribution across the two OSA risk subgroups. Both groups demonstrate an identical mean BMI of 30.1 kg/m^2^, with the majority of participants classified as overweight or obese, highlighting obesity as a shared predominant risk factor.

Participants meeting the screening criteria for obstructive sleep apnea–defined by the coexistence of habitual snoring and witnessed apneic episodes–were further stratified based on the presence or absence of spirometric airflow obstruction. The distribution of body mass index within these subgroups is illustrated in Figure 9 and summarized in Table 21.

Both OSA risk subgroups exhibited an identical mean BMI (30.1 kg/m^2^), reinforcing obesity as the principal common risk factor irrespective of the presence of airflow obstruction. In the OSA-with-obstruction subgroup, 23 of 27 participants with available BMI data (85.2%) were classified as overweight or obese, with no individuals in the underweight category. A similar pattern was observed in the OSA-without-obstruction subgroup (*n* = 78), where 82.1% were overweight or obese.

The subgroup with concurrent OSA risk and obstruction was older on average (55.6 vs. 50.0 years) and more predominantly male (67.9% vs. 61.5%). Additionally, 78.6% of these individuals were aged 46 years or older. This subgroup represents a potentially clinically relevant “triple-burden” screening phenotype, characterized by the coexistence of sleep-disordered breathing, airflow limitation, and excess adiposity, warranting prioritized and coordinated evaluation within pulmonology and sleep medicine frameworks.

#### 3.10.4. Additional Stratification by Age and BMI

**Figure 10:**
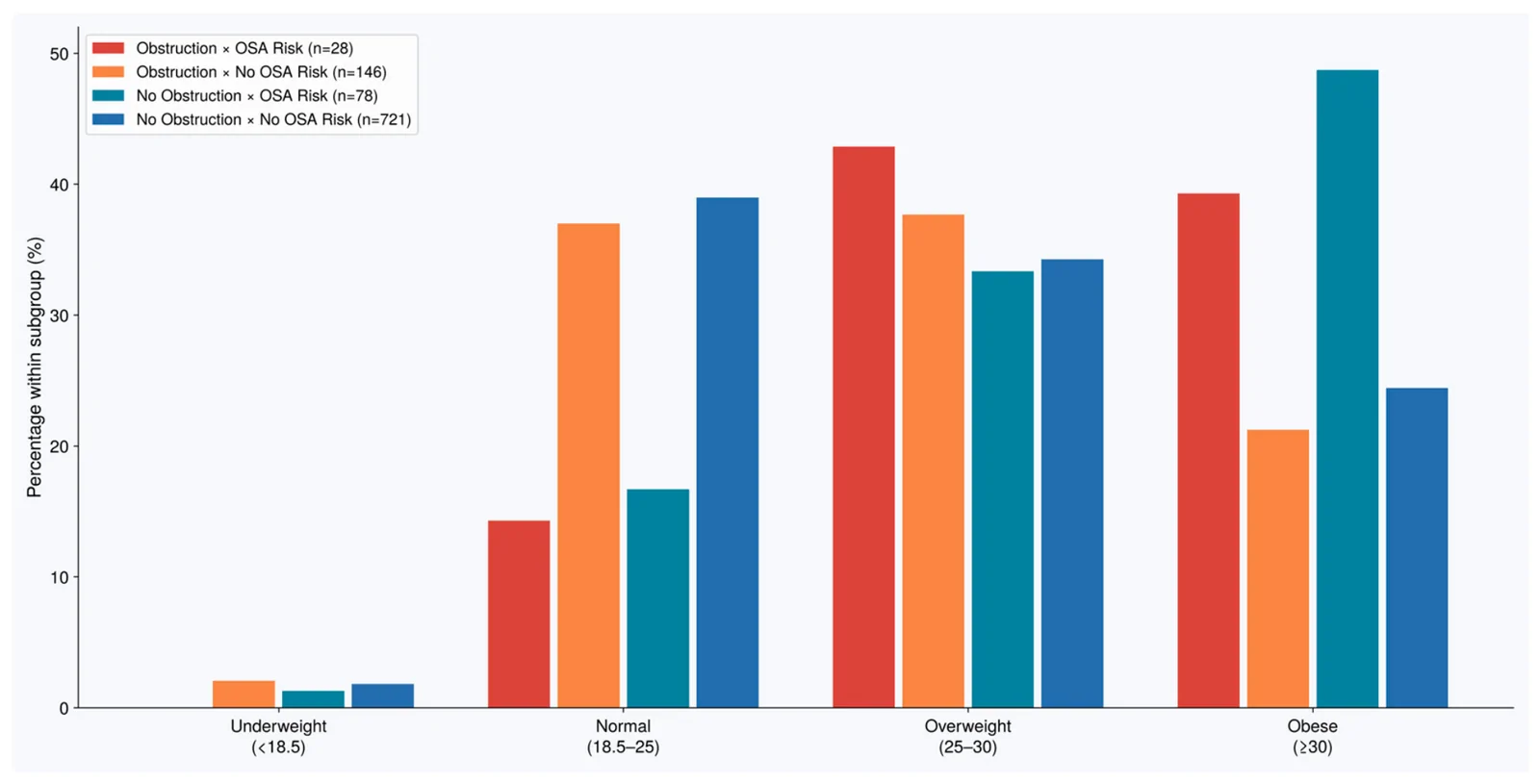
Matrix of BMI distribution across Obstruction × OSA Risk subgroups. Values represent the percentage of participants within each subgroup assigned to each BMI category. Subgroups with OSA risk consistently display higher obesity prevalence compared to those without OSA risk, regardless of obstruction status.

Figure 10 illustrates the distribution of BMI categories across the four combined subgroups defined by screening-detected pre-bronchodilator airflow obstruction and OSA risk status, while Table 22 and Table 23 provide a detailed breakdown of age group and BMI category distributions across all clinical subgroups.

The observed patterns confirm a consistent and robust association between obesity and OSA risk, independent of the presence of screening-detected pre-bronchodilator airflow obstruction. Participants with OSA risk exhibited markedly higher rates of obesity (40.7–48.7%) compared to those without OSA risk (approximately 20–30% across non-OSA subgroups), while mean BMI values were identical in the OSA subgroups (30.1 kg/m^2^), further supporting excess adiposity as the dominant shared risk factor.

In contrast, screening-detected pre-bronchodilator airflow obstruction alone was not independently associated with higher BMI categories, as reflected by relatively similar BMI distributions across obstructive and non-obstructive subgroups in the absence of OSA risk.

Age stratification (Table 22) demonstrates a clear shift toward older age groups among participants with screening-detected pre-bronchodilator airflow obstruction. Across all obstructive subgroups, the proportion of individuals aged ≥46 years was consistently high, with particularly pronounced aging in former smokers (59.5% aged >60 years) and in the symptomatic obstructive subgroup (83.7% aged ≥46 years). Even in the absence of symptoms, individuals with silent obstruction showed a substantial representation in older age categories (65.9% aged ≥46 years), underscoring the progressive and often under-recognized nature of airflow limitation.

Additionally, participants with concurrent OSA risk and obstruction displayed a predominantly middle-to-older age distribution, with 78.6% aged ≥46 years, reinforcing the clustering of cardiopulmonary and metabolic risk factors in this demographic.

Overall, these findings highlight two key patterns: (1) obesity is strongly linked to OSA risk regardless of spirometric status, and (2) screening-detected pre-bronchodilator airflow obstruction is predominantly associated with advancing age rather than BMI alone, supporting the need for integrated risk stratification incorporating both anthropometric and demographic factors.

## 4. Discussion

In this cross-sectional study of 979 adults across five coastal localities of the Mangalia municipality, a population characterized by marked demographic heterogeneity reflecting the seasonal influx of tourists and temporary workers alongside permanent residents, we found that screening-detected pre-bronchodilator airflow obstruction was identified in 17.9% of participants–approximately twice the rate of formally diagnosed CRD (9.3%)–with 15.7% of participants without a prior respiratory diagnosis demonstrating previously unrecognised airflow limitation. These figures are consistent with global estimates suggesting that a large proportion of obstructive lung disease remains undetected in community settings. Additionally, 10.9% of participants met the screening proxy criteria for elevated OSA risk, 35.2% were current smokers, and pneumococcal vaccination coverage was critically low at 3.4%.

Logistic regression analysis identified older age, lower FEV1/FVC ratio, and female sex as independent predictors of CRD diagnosis after adjustment for anthropometric variables. The association with female sex (OR 2.224) is consistent with the known predominance of asthma in adult women and aligns with the sex distribution observed in the diagnosed subgroups. The FEV1/FVC ratio as a significant predictor reinforces the diagnostic value of spirometry and supports its role as a screening tool in community settings. The modest overall model fit (Nagelkerke R^2^ = 0.079) is expected in a cross-sectional community-based study and reflects the multifactorial nature of CRD etiology, which extends beyond the variables captured in this analysis.

The expanded logistic regression analyses provide additional insight into the determinants of respiratory disease in this population. In Section 3.9.1, age and cumulative tobacco exposure (pack-years) emerged as the sole independent predictors of spirometric airflow obstruction, while current smoking status was not independently significant after adjustment, underscoring that the duration and intensity of exposure rather than current smoking behaviour are more closely related to obstructive physiology. In Section 3.9.2, BMI, current smoking, and older age were independent predictors of OSA screening risk, with female sex acting as a protective factor, consistent with the established male predominance of OSA; a locality-adjusted sensitivity analysis (Section 3.9.4) confirmed these associations but also found that locality remained statistically associated with OSA-risk after adjustment for the modelled individual-level covariates (highest in Olimp), indicating that site-level factors not captured by these covariates contribute meaningfully to this outcome. The absence of a significant association between CRD or obstruction and OSA risk after adjustment suggests that obesity accounts for much of the apparent relationship between these conditions. Section 3.9.3 showed that, among individuals with screening-detected pre-bronchodilator airflow obstruction, younger age and lower BMI were associated with remaining undiagnosed, suggesting a potential gap in early case detection among younger, leaner individuals. Respiratory symptoms were evaluated separately through the symptom × obstruction phenotype analysis and were retained as descriptive, hypothesis-generating categories rather than included as covariates in the obstruction regression model.

The overall CRD prevalence of 9.3% is broadly consistent with European epidemiological estimates. The observed COPD prevalence of 1.7% is lower than both national and European averages, which may reflect the relatively young mean age of the cohort (47.3 years) or, more plausibly, underdiagnosis consistent with patterns reported across Eastern Europe [4,6]. National surveys conducted by the Romanian Society of Pneumology in 2012 and 2017–2018 reported a COPD prevalence of 9.3%, with only 4% of patients formally diagnosed at the time of evaluation [7], a diagnostic gap strikingly similar to that observed in the present cohort. Furthermore, COPD prevalence estimates across Eastern European countries have been reported at 10–15% [7], against which our spirometric obstruction rate of 17.9% positions this coastal population at the higher end of the regional burden spectrum. By comparison, The BOLD study reported a global spirometry-confirmed COPD prevalence of 10.1% (stage II or higher, post-bronchodilator), substantially higher than self-reported figures–a discrepancy mirrored in our data where spirometric obstruction (17.9%) far exceeds diagnosed CRD (9.3%) [11]. The marked age gradient in CRD prevalence (6.1% in individuals under 40 years to 24.2% among those aged 71–80 years) is consistent with established epidemiological patterns across European populations [6]. The predominance of females among asthma patients (32 vs. 11 male cases; crude OR 1.935, 95% CI 1.230–3.045) aligns with known sex distributions in adult asthma, where hormonal and immunological factors contribute to female predominance after puberty [12]. The Romanian national CRD context, characterised by delayed guideline adoption and historically limited spirometry access in primary care [7], is consistent with the low diagnostic rates observed in this cohort.

The high prevalence of previously unrecognised airflow limitation (15.7% among individuals without a prior CRD diagnosis) is the most clinically actionable finding of this study. This figure is consistent with the pattern reported in the BOLD study and similar community screening initiatives, where spirometry-detected obstruction consistently exceeds self-reported diagnosis rates [11]. The low spirometry uptake (13.9% within the preceding six months) likely represents the primary structural driver of this diagnostic gap. Opportunistic spirometry–offered during routine primary care or pulmonology consultations, particularly for smokers and individuals aged ≥40 years–represents the most pragmatic intervention in this setting. It should be noted that pre-bronchodilator spirometry, as used in this study, may overestimate obstruction prevalence, and post-bronchodilator confirmation would be required before clinical diagnosis.

The proportion of participants meeting the two-item screening proxy criteria for elevated OSA risk (10.9%) reflects a symptom-based estimate that is not directly comparable to polysomnography-confirmed prevalence figures. Population-based studies using full polysomnography report substantially higher rates of sleep-disordered breathing–23.4% in women and 49.7% in men in the HypnoLaus cohort [13]–underscoring that symptom-based proxies capture only the most symptomatic segment of the at-risk population and likely underestimate true OSA burden in this cohort. The strong association with obesity (19.5% in obese vs. 7.9% in non-obese; absolute difference 11.6 percentage points; *p* < 0.001) is consistent with the findings of Alenezi et al., whose systematic review of 12 trials (*n* = 4095) reported OSA prevalence ranging from 12.6% to 88.9% in obese individuals, with BMI consistently and positively correlated with apnoea-hypopnoea index across studies [10]. The identical mean BMI of 30.1 kg/m^2^ in both OSA risk subgroups (with and without concurrent obstruction) reinforces obesity as the dominant shared risk factor across phenotypes. These findings support the integration of structured OSA screening–ideally using validated instruments such as STOP-BANG–into routine respiratory and primary care practice, particularly for obese individuals.

The prevalence of current smoking (35.2%) substantially exceeds the EU average of 18.4% for daily cigarette smokers recorded in the 2019 European Health Interview Survey (Eurostat EHIS, 2019) [14], consistent with Romania’s position among the Member States with the largest male-female smoking differentials in the EU, where men were 23.1 percentage points more likely than women to be daily smokers. The use of novel tobacco products (8.9%), particularly heated tobacco devices (5.7%), is consistent with patterns of elevated uptake observed in Central and Eastern European countries; a 2020 Eurobarometer analysis across 28 European nations reported ever HTP use ranging from 2.8% in France to 14.6% in the Czech Republic, with several CEE countries–including Bulgaria (12.0%), Latvia (13.8%) and Lithuania (10.6%)–substantially exceeding the EU average of 6.5% [15]. The comparable prevalence of novel tobacco use among individuals with and without CRDs suggests population-wide distribution rather than concentration in diagnosed patients. The long-term respiratory health effects of these products remain uncertain and require longitudinal investigation.

Several important gaps in healthcare delivery were identified. Spirometry utilisation was low (13.9%), pneumococcal vaccination coverage was minimal (3.4% overall; 5% in CRD-positive individuals vs. 3% in those without a diagnosis, *p* = 0.223), and only 5% of the overall population reported pulmonology follow-up. Among individuals with diagnosed CRDs, approximately 45% were under specialist care–suggesting that access improves substantially following diagnosis, but the primary challenge remains early detection. The finding that 15.7–17.6% of undiagnosed individuals reported dry cough or dyspnoea is consistent with a pattern of symptomatic individuals who have not yet been evaluated, and underscores the potential yield of opportunistic symptom-triggered spirometry in primary care. Cross-sectional design precludes causal inference; the associations described should be interpreted as risk correlations rather than directional relationships.

The cross-cutting clinical risk profile analysis further highlights the multidimensional nature of respiratory disease burden in this population. The symptomatic-with-obstruction subgroup (8.8%) represents the highest clinical burden, with approximately one-third of individuals reporting a prior CRD diagnosis and a markedly reduced mean FEV_1_/FVC (61.1%). The identification of a silent obstruction subgroup (9.0%; mean FEV_1_/FVC 64.2%; no respiratory symptoms) reinforces the need for systematic spirometric screening, as these individuals would not be detected through symptom-based approaches alone. Notably, both overt and silent obstruction are predominantly concentrated in individuals aged ≥46 years, which reinforces the age-dependent nature of airflow limitation.

The older age profile of former smokers further supports cumulative exposure effects. The higher rate of “any CRD” diagnosis among never-smokers is driven primarily by a higher asthma burden in this group: when disaggregated by subtype across the whole cohort, asthma was numerically more common among never-smokers than among current or former smokers (5.5% vs. 3.2% and 3.4%, respectively; Table 18), whereas COPD showed the expected positive association with tobacco exposure (0.2% in never-smokers vs. 2.6% in current and 4.0% in former smokers; Table 18). However, whereas the COPD gradient was statistically significant (χ^2^ = 12.84, df = 2, *p* = 0.0016), the whole-cohort asthma-by-smoking-status difference did not reach statistical significance (χ^2^ = 2.96, df = 2, *p* = 0.23) and should therefore be interpreted as a descriptive, hypothesis-generating pattern rather than an established explanatory mechanism. We additionally examined whether this asthma pattern reflects the sex composition of the smoking-status subgroups rather than a smoking-status-specific effect, since female sex was independently associated with CRD diagnosis in the adjusted model (Table 11; OR 2.224, 95% CI 1.158–4.270). Never-smokers in this cohort were disproportionately female relative to current and former smokers (60.4% vs. 47.8% and 35.4%, respectively; χ^2^ = 34.2, df = 2, *p* < 0.001; Table 20), and female sex remained an independent predictor of asthma diagnosis after adjustment for smoking status (adjusted OR 2.62, 95% CI 1.29–5.34, *p* = 0.008). Adjusting for sex did not strengthen the smoking-status–asthma association (current-vs-never-smoker OR 0.61 unadjusted vs. 0.63 sex-adjusted, both non-significant), indicating that the sex composition of the never-smoker subgroup may contribute to the observed asthma pattern rather than supporting a true smoking-status-specific protective effect. Because only 15 asthma cases occurred within the *n* = 173 obstructed subgroup, a sex-adjusted smoking-status model restricted to that subgroup would have been too sparse to yield a stable multivariable estimate; the sex-adjusted analysis was therefore conducted at the whole-cohort level, where this finding should be interpreted as suggestive of a contributing factor rather than as a confirmed explanation of the subgroup-specific pattern. Within the obstructed subgroup specifically (*n* = 173), the same qualitative direction was observed for asthma (12.3%, 5.4%, and 7.0% among never-, former-, and current-smokers, respectively; Table 19) but did not reach statistical significance at this smaller sample size (*p* = 0.45); the corresponding COPD (1.5%/10.8%/8.5%; *p* = 0.12) and chronic bronchitis (9.2%/5.4%/0%; *p* = 0.03, based on small cell counts) subtype distributions within this subgroup are presented descriptively in Table 19 given the limited statistical power of a three-way split of 173 participants. Taken together, these findings indicate that the apparent paradox reflects, at least in part, a difference in disease mix and sex composition rather than a robust, independently confirmed smoking-status effect on asthma risk; the COPD gradient with smoking exposure, by contrast, is well supported and consistent with expected pathophysiology.

Finally, the convergence of obesity, OSA risk, and screening-detected pre-bronchodilator airflow obstruction in the “triple-burden” subgroup with concurrent OSA risk and airflow obstruction (*n* = 28; mean BMI 30.1 kg/m^2^), of whom 11 (39.3%) were also obese, identifies a small but high-priority group. The consistently higher obesity prevalence across OSA risk subgroups, irrespective of obstruction, reinforces excess adiposity as the central shared risk factor and supports the need for integrated pulmonology and sleep medicine evaluation.

This study has several strengths. The relatively large sample size (*n* = 979), geographic coverage of all five localities within the Mangalia municipality, near-complete spirometry data availability (99.4%), and the inclusion of both traditional and novel respiratory risk factors represent meaningful epidemiological contributions in a setting with limited prior data. However, several limitations must be acknowledged. First, participation was voluntary and self-selected within a community screening campaign, introducing potential selection bias; health-aware individuals may be overrepresented relative to the general coastal population. Conversely, an outdoor beach-based recruitment setting could plausibly bias the sample in either direction–toward healthier, more mobile individuals, or toward those specifically seeking reassurance about symptoms–and the net direction of this bias cannot be determined from the available data. Findings should not be interpreted as population-representative prevalence estimates. Second, all chronic respiratory disease diagnoses were self-reported and were not verified against medical records or clinical examination findings. Specifically, chronic bronchitis, emphysema, COPD, and asthma were based entirely on participant recall of a prior clinician diagnosis. No CT or other chest imaging was performed during the campaign; therefore, the reported emphysema cases should not be interpreted as radiologically confirmed emphysema. CRD diagnoses were also not confirmed by spirometric disease staging or medical-record review. Medication and inhaled therapy use was also self-reported and was not independently verified against clinical records or prescription data, limiting its value as an objective indicator of established respiratory diagnosis or treatment. Third, spirometry was performed pre-bronchodilator only; this screening definition may overestimate obstruction in older adults because the FEV_1_/FVC ratio declines with age, and it does not account for lower-limit-of-normal reference values. A formal LLN-based (GLI-2012) sensitivity analysis could not be performed retrospectively, as ethnicity—a required input for the GLI-2012 reference equations—was not recorded as a study variable. Age-stratified obstruction estimates are therefore reported to demonstrate the strong age gradient in screening-detected obstruction. These findings should be interpreted as screening-detected airflow limitation rather than definitive spirometric confirmation of chronic obstructive pulmonary disease. Fourth, OSA risk was assessed using a two-item screening proxy rather than polysomnography or a validated multi-item questionnaire, limiting diagnostic precision. Fifth, the dynamic population composition of coastal localities–including seasonal workers and tourists actively recruited during the campaign period–may reduce the generalisability of findings to year-round residents, as the screened population may not reflect the permanent local demographic. Sixth, the cross-sectional design precludes causal inference. Seventh, population weighting was not applied, which may limit comparability with regional or national estimates. Eighth, missing data across some variables were handled by pairwise exclusion; principal-variable missingness is reported in Section 2.2 (spirometry: 0.6% missing) and alongside individual analyses. Ninth, between-locality comparisons are reported in Table 3 (demographics, CRD, smoking, obstruction, apnea risk, spirometry uptake, and vaccination, each with associated *p*-values); this table should be read alongside Table 16, Table 17, Table 18, Table 19, Table 20, Table 21, Table 22 and Table 23 as providing descriptive locality- and subgroup-level context. The cross-tabulations in Table 16, Table 17, Table 18, Table 19, Table 20 and Table 21 and the age/BMI distributions in Table 22 and Table 23 remain univariate and exploratory, however, and are not adjusted for confounding; they should therefore be interpreted as hypothesis-generating rather than confirmatory, complementing rather than duplicating the adjusted multivariable models presented in Table 11, Table 12, Table 13 and Table 14. Tenth, the 21 formal significance tests reported in the descriptive comparison tables (Table 3′s locality comparisons and Table 7’s healthcare-utilisation/vaccination comparisons) were subjected to post hoc Benjamini-Hochberg FDR correction for multiple comparisons (Table 10); 10 of 11 nominally significant findings remained significant after adjustment. Table 4, Table 5, Table 6, Table 9, Table 16, Table 17, Table 18, Table 19, Table 20, Table 21, Table 22 and Table 23 present purely descriptive counts and percentages without accompanying formal hypothesis tests and are therefore not subject to a multiplicity concern; these should nonetheless be interpreted as exploratory, complementing rather than duplicating the pre-specified multivariable models (Table 11, Table 12, Table 13 and Table 14), which constitute the primary inferential analyses of this report. Eleventh, the questionnaire captured only three comorbidities (cardiovascular disease, diabetes, stroke); clinically relevant conditions known to influence respiratory symptoms and diagnostic accuracy–including bronchiectasis, gastro-oesophageal reflux disease, hypertension, thyroid disorders, and mental health conditions–were not systematically captured and represent a further source of potential residual confounding not addressed in the present analysis. A composite phenotype-based model jointly incorporating symptom burden, smoking, obesity, and OSA-risk as simultaneous predictors of a pre-specified ‘severe obstruction’ outcome was beyond the descriptive scope of the present report and is proposed as a direction for future analysis using the pooled multi-year dataset. Twelfth, formal calibration assessment (e.g., Hosmer–Lemeshow test) was not performed for any of the four multivariable models; while discrimination-related diagnostics (linearity in the logit, variance inflation factors) were assessed and satisfactory, calibration should be examined in future analyses of the pooled multi-year dataset. Thirteenth, the CRD subtype-by-smoking-status disaggregation within the obstructed subgroup (Table 19, *n* = 173) is based on small cell counts (several subtype-by-group cells < 5 participants), so subtype-level percentages in this specific subgroup should be interpreted as exploratory rather than confirmatory, even where a nominal *p*-value is below 0.05. Fourteenth, because Table 3 showed significant between-locality differences in age, sex distribution, and current-smoking prevalence—each a covariate in the principal multivariable models (Table 11, Table 12, Table 13 and Table 14)—we conducted a locality-adjusted sensitivity analysis (Section 3.9.4, Table 15). Locality was not a statistically significant covariate for CRD diagnosis, airflow obstruction, or diagnosis within the obstructed subgroup, and did not materially change the principal covariate estimates in any of these three models. For OSA-risk, locality remained statistically significant after adjustment (*p* = 0.038), reflecting site-level variation (highest in Olimp) beyond the modelled individual predictors; however, the age, sex, BMI, and current-smoking associations with OSA-risk were not materially altered by this adjustment. These findings indicate that the reported associations are generally robust to locality-level clustering, with the partial exception of OSA-risk, for which locality remained statistically associated with the outcome beyond the modelled individual-level covariates.

## 5. Conclusions

The prevalence estimates reported here should be interpreted in light of the self-selected, non-random recruitment strategy and the use of a fixed pre-bronchodilator obstruction threshold detailed in Section 4, and are best understood as proportions observed among screening participants rather than population-representative estimates.

This study provides the first multi-locality epidemiological characterisation of respiratory health in the Mangalia coastal area, encompassing 979 adults. The central finding is that one in six adults without a previous respiratory diagnosis demonstrated spirometric airflow limitation, suggesting that opportunistic spirometry performed in primary care or pulmonology settings could substantially improve case detection in Romanian coastal communities. The overall prevalence of diagnosed CRDs was 9.3%, increasing markedly with age to 24.2% among individuals aged 71–80 years. Multivariable logistic regression identified older age (OR 1.022, 95% CI 1.001–1.041), reduced FEV_1_/FVC ratio (OR 0.958, 95% CI 0.936–0.981), and female sex (adjusted OR 2.224, 95% CI 1.158–4.270) as independent predictors of CRD diagnosis.

Cross-cutting analysis of clinical risk profiles identified four symptomatology-based subgroups, highlighting both the high-burden symptomatic-with-obstruction group (*n* = 86; CRD rate 32.6%) and a clinically silent obstruction subgroup (*n* = 88; 9.0%) that would be missed by symptom-based screening alone. Smoking status analysis showed that never-smokers with obstruction had the highest CRD diagnosis rate (24.6%), a pattern that may partly reflect the higher asthma burden and differing sex composition of this subgroup, although the smoking-status–asthma association was not independently confirmed, as detailed in Section 4. Additionally, a triple-burden subgroup with concurrent OSA risk and airflow obstruction (*n* = 28; mean BMI 30.1 kg/m^2^), of whom 11 (39.3%) were also obese, was identified as a high-priority group for specialist referral.

A considerable proportion of participants met the prespecified two-item screening proxy for elevated OSA probability (10.9%), which was strongly associated with obesity. Tobacco exposure remains a major public health concern, with over one-third of participants reporting current smoking and 8.9% using novel tobacco products. Preventive care gaps were evident, particularly the critically low pneumococcal vaccination coverage (3.4%).

These findings support a unified community respiratory health strategy incorporating: (1) opportunistic pre-bronchodilator spirometry with post-bronchodilator confirmation for screen-positive individuals in primary care, targeting smokers and adults aged ≥40 years; (2) structured OSA risk stratification–using validated instruments in obese individuals; (3) active promotion of pneumococcal vaccination, particularly among CRD-diagnosed patients where uptake (5%) remains critically below recommended targets; and (4) surveillance of novel tobacco product use given rapidly evolving exposure patterns. Future research should incorporate longitudinal follow-up, post-bronchodilator spirometry confirmation, polysomnographic OSA validation, and population-based sampling to refine disease burden estimates and support targeted intervention planning.

## Figures and Tables

**Table 1:** Missing-data summary for the principal cross-sectional variable (spirometry) and for each of the four multivariable regression models (Section 3.9), with the reason for missingness where documented in the source data. Eligible *n* reflects the denominator available before listwise exclusion for that model; descriptive comparisons elsewhere in the manuscript (Section 3.1, Section 3.2, Section 3.3, Section 3.4, Section 3.5, Section 3.6, Section 3.7 and Section 3.8) use pairwise/available-case analysis and are not listed here. For Models 2–4, missingness reflects isolated gaps across multiple covariates rather than a single documented cause and was not itemised by individual field in the source dataset.

Analysis/Model	Eligible *n*	Excluded (Missing)	Analysis *n*	% Missing	Reason for Missingness
Spirometry (FEV_1_/FVC ratio)	979	6	973	0.6%	Medical contraindication or patient declined (*n* = 2); spirometry result undocumented/unlocated (*n* = 1); test apparently not completed, no reason recorded (*n* = 1); FEV1/FVC missing (*n* = 2)
Sex (sex-stratified analyses)	979	2	977	0.2%	Sex not recorded for 2 participants
Age (age-group stratification)	979	3	976	0.3%	Age missing/unusable for 3 participants (2 questionnaires had a non-numeric note entered in the age field instead of a value; 1 was left blank)
Model 1 (Section 3.9)—predictors of CRD diagnosis (age, FEV_1_/FVC, height, weight, sex)	979	10	969	1.0%	6 as above, plus isolated blank or non-numeric entries for a single anthropometric or demographic field (height, weight, or age) in 4 additional questionnaires, with no reason documented
Model 2 (Section 3.9.1) —predictors of airflow obstruction (9 covariates)	973	8	965	0.8%	Isolated missing values in one or more covariates (BMI components, pack-years, or exposure items); not separately itemised by field in the source dataset
Model 3 (Section 3.9.2) —predictors of OSA screening risk (6 covariates)	979	14	965	1.4%	As above
Model 4 (Section 3.9.3) —predictors of prior diagnosis among obstructed subgroup (age, sex, BMI)	174	2	172	1.1%	Isolated missing BMI or age value; not separately itemised

**Table 2:** Summary of the principal results across three domains: demographic characteristics, respiratory disease burden, and key risk indicators, including airflow obstruction, sleep apnea risk, smoking prevalence, and vaccination coverage in the study population.

Demographics	Respiratory Disease	Key Risk Signals
979 enrolled (all included)	Any CRD: 9.3% (*n* = 91)	Obstruction FEV1/FVC < 70%: 17.9%
Female 51.4%/Male 48.4%	Asthma: 4.4% · Bronchitis: 3.0%	Sleep Apnea At-Risk: 10.9% (*n* = 107)
Mean age: 47.3 ± 13.5 yrs (18–82)	COPD: 1.7% · Emphysema: 0.3%	Undiagnosed obstruction: 15.7%
Largest group: 41–60 yrs (*n* = 497)	CRD rises with age: 6.1% → 24.2%	Mean BMI: 27.1
5 localities: 147–257 per site	Only 14% had spirometry	Obese: 26.3%
Cardiac comorbidity: 19.7%	Current smokers: 35.2%	Pneumococcal vaccine: only 3.4%

**Table 3:** Study population characteristics by locality (*n* = 979). Distribution of demographic variables, respiratory disease prevalence, risk factors, and healthcare indicators across the five coastal localities, with corresponding statistical comparisons (*p*-values).

Variable	Neptun (*n* = 147)	Olimp (*n* = 178)	Saturn (*n* = 187)	Mangalia (*n* = 257)	Jupiter (*n* = 210)	*p*-Value	Total (*n* = 979)
Female, *n* (%)	88 (60%)	88 (49%)	90 (48%)	143 (56%)	94 (45%)	*p* = 0.028	503 (51%)
Age, mean ± SD (yrs)	50.8 ± 13.5	47.4 ± 12.5	49.3 ± 13.8	47.1 ± 14.9	43.1 ± 11.3	*p* < 0.001	47.3 ± 13.5
Any CRD, *n* (%)	19 (13%)	13 (7%)	19 (10%)	26 (10%)	14 (7%)	*p* = 0.265	91 (9%)
Current smoker, *n* (%)	42 (29%)	71 (40%)	50 (27%)	109 (42%)	73 (35%)	*p* = 0.003	345 (35%)
Mean BMI	27.3	26.4	27.2	26.8	27.7	*p* = 0.149	27.1
Obese BMI ≥ 30, *n* (%)	43 (29%)	36 (20%)	48 (26%)	64 (25%)	66 (31%)	*p* = 0.125	257 (26%)
FEV1/FVC < 70%, *n* (%)	29/144 (20%)	36/175 (21%)	27/187 (14%)	50/257 (19%)	32/210 (15%)	*p* = 0.411	174/973 (18%)
Apnea risk, *n* (%)	18 (12%)	28 (16%)	20 (11%)	26 (10%)	15 (7%)	*p* = 0.101	107 (11%)
Spirometry past 6mo, *n* (%)	17 (12%)	11 (6%)	26 (14%)	45 (18%)	37 (18%)	*p* = 0.005	136 (14%)
Influenza vaccine, *n* (%)	48 (33%)	36 (20%)	37 (20%)	49 (19%)	26 (12%)	*p* < 0.001	196 (20%)
Pneumococcal vaccine, *n* (%)	7 (5%)	7 (4%)	4 (2%)	12 (5%)	3 (1%)	*p* = 0.224	33 (3%)
Pulm. follow-up, *n* (%)	13 (9%)	7 (4%)	9 (5%)	15 (6%)	5 (2%)	*p* = 0.077	49 (5%)

**Table 4:** Prevalence of chronic respiratory diseases by sex and age group. Distribution of bronchitis, emphysema, COPD, asthma, and overall CRD prevalence across sex and age strata, highlighting age-related increases and sex differences in disease patterns.

Diagnosis	Total *n* (%)	Female	Male	≤40 yrs (*n* = 312)	41–60 yrs (*n* = 497)	>60 yrs (*n* = 167)
Bronchitis	29 (3.0%)	17	12	6 (1.9%)	14 (2.8%)	9 (5.4%)
Emphysema	3 (0.3%)	2	1	0 (0%)	3 (0.6%)	0 (0%)
COPD	17 (1.7%)	10	7	1 (0.3%)	8 (1.6%)	8 (4.8%)
Bronchial Asthma	43 (4.4%)	32	11	12 (3.8%)	20 (4.0%)	11 (6.6%)
Any CRD	91 (9.3%)	60F (11.9%)	31M (6.5%)	19 (6.1%)	44 (8.9%)	28 (16.8%)
No Diagnosis	888 (90.7%)	443F (88.1%)	443M (93.5%)	293 (93.9%)	453 (91.1%)	139 (83.2%)

**Table 5:** Smoking status and novel tobacco use by diagnostic group. Distribution of smoking history (never, current, former, and ever smokers) and use of novel tobacco products (e-cigarettes, heated tobacco, hookah, and nicotine pouches) across participants with chronic respiratory disease, undiagnosed individuals, and those with spirometric airflow obstruction. Smoking-status classification was unavailable for 1 of the 174 participants with obstruction (173/174 classified); 5 participants overall could not be classified into the never/current/former categories. Of these 5, two have a chronic respiratory disease diagnosis, so the Any CRD column totals sum to 89 rather than 91.

Smoking Variable	Any CRD (*n* = 91)	No Diagnosis (*n* = 888)	Obstruction (*n* = 174)	Total (*n* = 979)
Never smoker, *n* (%)	41 (45%)	413 (47%)	65 (37%)	454 (46%)
Current smoker, *n* (%)	29 (32%)	316 (36%)	71 (41%)	345 (35%)
Former smoker, *n* (%)	19 (21%)	156 (18%)	37 (21%)	175 (18%)
Ever smoked (current + former)	48 (53%)	472 (53%)	108 (62%)	520 (53%)
E-cigarette use, *n* (%)	4 (4%)	33 (4%)	3 (2%)	37 (3.8%)
Heated tobacco (IQOS), *n* (%)	4 (4%)	52 (6%)	9 (5%)	56 (5.7%)
Hookah, *n* (%)	1 (1%)	9 (1%)	2 (1%)	10 (1.0%)
Nicotine pouches, *n* (%)	0 (0%)	2 (0%)	0 (0%)	2 (0.2%)
Any novel tobacco, *n* (%)	8 (8.9%)	79 (9%)	13 (7%)	87 (9%)

**Table 6:** Sex-stratified snoring and witnessed apnoea rates, and OSA risk indicators by obesity status and presence of sleep modifications (*n* = 979). Sleep modifications defined as self-reported insomnia or short sleep duration (3–4 h/night). Snoring and witnessed apneas were highest in obese individuals with concurrent sleep disturbances. The non-obese comparator group (*n* = 722) includes 4 participants with incomplete anthropometric data who could not be confirmed as obese.

Group	*n*	Snoring	Witnessed Apneas	Sleep Modifications
Female (*n* = 503)	503	246 (48.9%)	50 (9.9%)	—
Male (*n* = 474)	474	321 (67.7%)	71 (15.0%)	—
Obese + sleep modifications (*n* = 70)	70	56 (80.0%)	22 (31.4%)	Insomnia and/or short sleep
Obese + no sleep modifications (*n* = 187)	187	144 (77.0%)	32 (17.1%)	None reported
Non-obese (*n* = 722)	722	367 (50.8%)	67 (9.3%)	—

**Table 7:** Healthcare utilisation and vaccination status by diagnostic group. Comparison of spirometry uptake, healthcare use (emergency visits, hospitalisations, and follow-up care), functional limitation, and vaccination coverage across participants with chronic respiratory disease, undiagnosed individuals, and those with spirometric airflow obstruction, including corresponding statistical comparisons.

Indicator	Any CRD (*n* = 91)	No Diagnosis (*n* = 888)	Obstruction (*n* = 174)	Total (*n* = 979)
Spirometry past 6 months	40 (44%)	96 (11%)	39 (22%)	136 (14%) *p* < 0.001
Any ER visit (past year)	9 (10%)	31 (3%)	7 (4%)	40 (4%) *p* = 0.008
Any hospitalisation (past year)	5 (5%)	6 (1%)	4 (2%)	11 (1%) *p* = 0.002
GP follow-up	26 (29%)	41 (5%)	17 (10%)	67 (7%) *p* < 0.001
Pulmonologist follow-up	41 (45%)	8 (1%)	23 (13%)	49 (5%) *p* < 0.001
Activity limited by disease	35 (38%)	35 (4%)	22 (13%)	70 (7%) *p* < 0.001
Influenza vaccine	20 (22%)	176 (20%)	32 (18%)	196 (20%) *p* = 0.724
Pneumococcal vaccine	5 (5%)	28 (3%)	7 (4%)	33 (3.4%) *p* = 0.223
COVID-19 vaccine	54 (59%)	558 (63%)	115 (66%)	612 (62.5%) *p* = 0.587

**Table 8:** Healthcare utilisation and vaccination coverage by CRD diagnostic status: previously known CRD (*n* = 91) versus newly identified at screening (*n* = 139, defined as spirometric obstruction FEV_1_/FVC < 70% with no prior CRD diagnosis). The stark contrast in pulmonologist follow-up (45.1% vs. 1.4%) and activity limitation (38.5% vs. 4.3%) reinforces the diagnostic gap and its functional consequences.

Indicator	Previously Known CRD (*n* = 91)	Newly Identified at Screening (*n* = 139)
Spirometry past 6 months	40 (44.0%)	20 (14.4%)
ER visit past year	9 (9.9%)	3 (2.2%)
Hospitalization past year	5 (5.5%)	2 (1.4%)
GP follow-up	26 (28.6%)	9 (6.5%)
Pulmonologist follow-up	41 (45.1%)	2 (1.4%)
Influenza vaccine	20 (22.0%)	23 (16.5%)
Pneumococcal vaccine	5 (5.5%)	3 (2.2%)
COVID-19 vaccine	54 (59.3%)	92 (66.2%)
Activity limited by disease	35 (38.5%)	6 (4.3%)

**Table 9:** Allergy prevalence by self-reported diagnosis category (*n* = 75). Self-reported emphysema (*n* = 3) is included for completeness but is underpowered for statistical comparison. Allergy burden was highest in the self-reported asthma group (67% any allergy), consistent with the known atopic component of asthma pathogenesis.

Self-Reported Diagnosis	*n*	Any Allergy	Dust	Pollen	Mould
Self-reported chronic bronchitis	29	14 (48%)	5 (17%)	3 (10%)	3 (10%)
Self-reported asthma	43	29 (67%)	14 (33%)	15 (35%)	10 (23%)
Self-reported emphysema	3	0 (0%)	0 (0%)	0 (0%)	0 (0%)

**Table 10:** Sensitivity Analysis–Benjamini-Hochberg FDR Correction for Multiple Comparisons (21 tests across Table 3 and Table 7).

Source	Comparison	Raw *p*	FDR-adj. *p*	Sig. (adj.)
Table 3	Age, mean ± SD	<0.001	0.004	Yes
Table 3	Female, *n* (%)	0.028	0.053	No
Table 3	Current smoker, *n* (%)	0.003	0.008	Yes
Table 3	Mean BMI	0.149	0.209	No
Table 3	Obese BMI ≥ 30, *n* (%)	0.125	0.188	No
Table 3	Any CRD, *n* (%)	0.265	0.309	No
Table 3	FEV1/FVC < 70%, *n* (%)	0.411	0.454	No
Table 3	Apnea risk, *n* (%)	0.101	0.163	No
Table 3	Spirometry past 6mo, *n* (%)	0.005	0.012	Yes
Table 3	Influenza vaccine, *n* (%)	<0.001	0.004	Yes
Table 3	Pneumococcal vaccine, *n* (%)	0.224	0.277	No
Table 3	Pulm. follow-up, *n* (%)	0.077	0.135	No
Table 7	Spirometry past 6 months	<0.001	0.004	Yes
Table 7	Any ER visit (past year)	0.008	0.017	Yes
Table 7	Any hospitalisation (past year)	0.002	0.006	Yes
Table 7	GP follow-up	<0.001	0.004	Yes
Table 7	Pulmonologist follow-up	<0.001	0.004	Yes
Table 7	Activity limited by disease	<0.001	0.004	Yes
Table 7	Influenza vaccine	0.724	0.724	No
Table 7	Pneumococcal vaccine	0.223	0.277	No
Table 7	COVID-19 vaccine	0.587	0.616	No

**Table 11:** Logistic Regression Analysis–Predictors of Chronic Respiratory Disease Diagnosis (*n* = 969).

Variable	OR	95% CI	*p*
Age (per year)	1.022	1.001–1.041	0.028
FEV1/FVC ratio (per unit)	0.958	0.936–0.981	<0.001
Height (per cm)	0.990	0.954–1.028	0.580
Weight (per kg)	1.011	0.995–1.027	0.163
Sex (Female vs. Male)	2.224	1.158–4.270	0.016

**Table 12:** Logistic Regression Analysis–Predictors of Spirometric Airflow Obstruction (*n* = 965). Dependent variable: FEV_1_/FVC < 70%. Nagelkerke R^2^ = 0.131.

Variable	OR	95% CI	*p*
Age (per year)	1.054	1.038–1.071	<0.001
Sex (Female vs. Male)	0.798	0.554–1.149	0.225
BMI (per unit)	0.97	0.933–1.008	0.115
Pack-years (per year)	1.013	1.002–1.024	0.025
Current smoker (vs. never/former)	1.343	0.877–2.057	0.175
Novel tobacco use	1.093	0.547–2.185	0.801
Pneumococcal vaccination	0.743	0.293–1.88	0.530
OSA risk (screening proxy)	1.411	0.844–2.36	0.189
Occupational exposure	0.723	0.241–2.168	0.562

**Table 13:** Logistic Regression Analysis–Predictors of OSA Screening Risk (*n* = 965). Dependent variable: co-reporting of habitual snoring and witnessed apnoeic episodes. Nagelkerke R^2^ = 0.12.

Variable	OR	95% CI	*p*
Age (per year)	1.023	1.005–1.041	0.014
Sex (Female vs. Male)	0.608	0.392–0.943	0.026
BMI (per unit)	1.115	1.072–1.16	<0.001
Current smoker	1.958	1.259–3.045	0.003
Any CRD diagnosis	1.311	0.671–2.559	0.428
Airflow obstruction (FEV_1_/FVC < 70%)	1.305	0.77–2.213	0.322

**Table 14:** Logistic Regression Analysis–Predictors of CRD Diagnosis Among Participants with Airflow Obstruction (*n* = 172), Reduced Parsimonious Model. Dependent variable: presence of a prior CRD diagnosis. Model restricted to age, sex, and BMI given limited events (35 diagnosed cases; events-per-variable ≈11.7). Nagelkerke R^2^ = 0.108.

Variable	OR	95% CI	*p*
Age (per year)	1.055	1.018–1.094	0.004
Sex (Female vs. Male)	2.184	0.971–4.914	0.059
BMI (per unit)	1.100	1.009–1.198	0.030

**Table 15:** Sensitivity analysis: locality-adjusted refits of the four principal multivariable models (Table 11, Table 12, Table 13 and Table 14), fitted on the same analytic samples as the original models (*n* = 969, 965, 965, and 172, respectively). Locality entered as a fixed categorical covariate (5 levels, reference: Mangalia); likelihood-ratio test of the joint locality term against the corresponding base model.

Model (Table)	Outcome	*n*	Locality LR χ^2^ (df = 4)	*p*	Effect on Principal Covariates
8	CRD diagnosis	969	3.44	0.486	No material change
9	Airflow obstruction	965	3.64	0.457	No material change
10	OSA-risk	965	10.18	0.038	Locality block significant (driven by Olimp); principal covariates materially unchanged
11	Diagnosis within obstructed subgroup	172	5.14	0.274	No material change (Neptun nominally elevated, underpowered)

**Table 16:** Characteristics of subgroups defined by symptomatology and airflow obstruction.

Subgroup	*n* (%)	Mean Age (yrs)	Female (%)	Any CRD (%)	Mean FEV1/FVC (%)	Mean BMI
Symptomatic + Obstruction	86 (8.8%)	56.7 ± 12.9	53.5%	32.6%	61.1 ± 10.4	27.7
Symptomatic without Obstruction	334 (34.3%)	46.7 ± 13.7	60.8%	10.8%	79.1 ± 6.1	27.5
Asymptomatic without Obstruction	465 (47.8%)	45.0 ± 12.6	46.9%	4.3%	79.5 ± 6.2	26.7
Asymptomatic + Obstruction (Silent)	88 (9.0%)	51.9 ± 12.9	37.5%	8.0%	64.2 ± 5.3	26.9

**Table 17:** Characteristics of subgroups defined by smoking status and screening-detected pre-bronchodilator airflow obstruction. Smoking status could not be classified for 1 participant with obstruction. BMI-category breakdowns for the Active Smokers + Obstruction subgroup in Section 3.10.4 are based on the 70 participants with complete anthropometric data (1 participant is missing weight); age-category breakdowns in Section 3.10.4 use the full *n* = 71, as age was recorded for all participants in this subgroup.

Subgroup	*n* (%)	Mean Age (yrs)	Female (%)	Any CRD (%)	Mean FEV1/FVC (%)	Mean BMI	OSA Risk
Active Smokers + Obstruction	71 (7.3%)	53.2 ± 12.4	47.1%	15.5%	63.0 ± 7.8	27.1	17 (23.9%)
Former Smokers + Obstruction	37 (3.8%)	61.5 ± 9.7	29.7%	21.6%	62.5 ± 6.4	28.1	5 (13.5%)
Never-Smokers + Obstruction	65 (6.7%)	51.6 ± 13.5	53.8%	24.6%	62.3 ± 10.0	27.1	6 (9.2%)

**Table 18:** Asthma and COPD diagnosis by smoking status, whole cohort (*n* = 974; smoking status unclassified for 5 participants). χ^2^ test of independence; *p*-values confirmed by exact permutation testing given small expected cell counts for COPD. Column totals for asthma sum to one fewer than the 43 cases reported for the whole cohort (Section 3.2), reflecting one asthma case among the participants with unclassified smoking status.

CRD Subtype	Never (*n* = 454)	Former (*n* = 175)	Current (*n* = 345)	χ^2^ (df = 2)	*p*-Value
Asthma, *n* (%)	25 (5.5%)	6 (3.4%)	11 (3.2%)	2.96	0.23
COPD, *n* (%)	1 (0.2%)	7 (4.0%)	9 (2.6%)	12.84	0.0016

**Table 19:** CRD subtype by smoking status within the screening-detected obstructed subgroup (FEV_1_/FVC < 70%, *n* = 173). * Exact (Monte Carlo permutation) *p*-values, given multiple expected cell counts < 5 in this subgroup; findings should be interpreted as exploratory (see Section 4, Limitations).

CRD Subtype	Never (*n* = 65)	Former (*n* = 37)	Current (*n* = 71)	*p*-Value *
Asthma, *n* (%)	8 (12.3%)	2 (5.4%)	5 (7.0%)	0.45
COPD, *n* (%)	1 (1.5%)	4 (10.8%)	6 (8.5%)	0.12
Chronic bronchitis, *n* (%)	6 (9.2%)	2 (5.4%)	0 (0.0%)	0.03

**Table 20:** Sex distribution across smoking-status groups, whole cohort (*n* = 974). χ^2^ = 34.2, df = 2, *p* < 0.001.

Smoking Status	*n*	Female, *n* (%)	Male, *n* (%)
Never-smoker	454	274 (60.4%)	180 (39.6%)
Former smoker	175	62 (35.4%)	113 (64.6%)
Current smoker	345	165 (47.8%)	180 (52.2%)

**Table 21:** Characteristics of subgroups defined by OSA risk and airflow obstruction. Of the 107 participants meeting OSA-risk criteria, 1 lacked spirometry data and could not be classified by obstruction status; the two rows therefore sum to 106.

Subgroup	*n* (%)	Mean Age (yrs)	Female (%)	Any CRD (%)	Mean FEV1/FVC (%)	Mean BMI
OSA Risk + Obstruction	28 (2.9%)	55.6 ± 11.0	32.1%	21.4%	63.2 ± 5.3	30.1 ± 5.5
OSA Risk without Obstruction	78 (8.0%)	50.0 ± 12.4	38.5%	10.3%	78.2 ± 5.6	30.1 ± 6.4

**Table 22:** Age group distribution across clinical subgroups. Age was missing for 1 participant in the Asymptomatic without Obstruction subgroup; age-category counts therefore sum to 464 rather than 465.

Subgroup	*n*	18–30 yrs	31–45 yrs	46–60 yrs	>60 yrs
Symptomatic + Obstruction	86	3 (3.5%)	11 (12.8%)	36 (41.9%)	36 (41.9%)
Symptomatic without Obstruction	334	46 (13.8%)	108 (32.3%)	126 (37.7%)	54 (16.2%)
Asymptomatic without Obstruction	465	73 (15.7%)	157 (33.8%)	185 (39.8%)	49 (10.5%)
Asymptomatic + Obstruction (Silent)	88	6 (6.8%)	24 (27.3%)	32 (36.4%)	26 (29.5%)
Active Smokers + Obstruction	71	6 (8.5%)	12 (16.9%)	31 (43.7%)	22 (31.0%)
Former Smokers + Obstruction	37	0 (0.0%)	3 (8.1%)	12 (32.4%)	22 (59.5%)
Never-Smokers + Obstruction	65	3 (4.6%)	20 (30.8%)	25 (38.5%)	17 (26.2%)
OSA Risk + Obstruction	28	0 (0.0%)	6 (21.4%)	12 (42.9%)	10 (35.7%)
OSA Risk without Obstruction	78	7 (9.0%)	19 (24.4%)	36 (46.2%)	16 (20.5%)

**Table 23:** BMI category distribution across clinical subgroups. Percentages for BMI categories are calculated among participants with available BMI data; subgroup *n* denotes the total subgroup size. BMI was unavailable for 1 participant in each of the Symptomatic + Obstruction, Asymptomatic + Obstruction, Never-Smokers + Obstruction, and OSA Risk + Obstruction subgroups, and for 1 participant in the Active Smokers + Obstruction subgroup.

Subgroup	*n*	Underweight (<18.5)	Normal (18.5–25)	Overweight (25–30)	Obese (≥30)	Mean BMI (kg/m^2^)
Symptomatic + Obstruction	86	1 (1.2%)	28 (32.9%)	32 (37.6%)	24 (28.2%)	27.7 ± 5.1
Symptomatic without Obstruction	334	6 (1.8%)	119 (35.6%)	109 (32.6%)	100 (29.9%)	27.5 ± 5.7
Asymptomatic without Obstruction	465	8 (1.7%)	179 (38.5%)	164 (35.3%)	114 (24.5%)	26.7 ± 4.7
Asymptomatic + Obstruction (Silent)	88	2 (2.3%)	30 (34.5%)	37 (42.5%)	18 (20.7%)	26.9 ± 4.3
Active Smokers + Obstruction	71	3 (4.3%)	25 (35.7%)	25 (35.7%)	17 (24.3%)	27.1 ± 5.4
Former Smokers + Obstruction	37	0 (0.0%)	8 (21.6%)	19 (51.4%)	10 (27.0%)	28.1 ± 4.0
Never-Smokers + Obstruction	65	0 (0.0%)	25 (39.1%)	24 (37.5%)	15 (23.4%)	27.1 ± 4.4
OSA Risk + Obstruction	28	0 (0.0%)	4 (14.8%)	12 (44.4%)	11 (40.7%)	30.1 ± 5.5
OSA Risk without Obstruction	78	1 (1.3%)	13 (16.7%)	26 (33.3%)	38 (48.7%)	30.1 ± 6.4

## Data Availability

The data presented in this study are available on request from the corresponding author. The data are not publicly available due to privacy and ethical restrictions in accordance with GDPR and Romanian Law 190/2018.

## Abbreviations

The following abbreviations are used in this manuscript:

BMI	Body Mass Index
BOLD	Burden of Obstructive Lung Disease
CAT	COPD Assessment Test
CI	Confidence Interval
COPD	Chronic Obstructive Pulmonary Disease
CRD	Chronic Respiratory Disease
EHIS	European Health Interview Survey
ER	Emergency Room
EU	European Union
FEV_1_	Forced Expiratory Volume in 1 s
FVC	Forced Vital Capacity
GOLD	Global Initiative for Chronic Obstructive Lung Disease
GP	General Practitioner
HTP	Heated Tobacco Products
OR	Odds Ratio
OSA	Obstructive Sleep Apnea
SD	Standard Deviation
STOP-BANG	Snoring, Tiredness, Observed apnea, Pressure, BMI, Age, Neck, Gender

## References

[1] Chen X., Zhou C.-W., Fu Y.-Y., Li Y.-Z., Chen L., Zhang Q.-W., Chen Y.-F. (2023). Global, regional, and national burden of chronic respiratory diseases and associated risk factors, 1990–2019: Results from the Global Burden of Disease Study 2019. Front. Med..

[2] Momtazmanesh S., Moghaddam S.S., Ghamari S.-H., Rad E.M., Rezaei N., Shobeiri P., Aali A., Abbasi-Kangevari M., Abbasi-Kangevari Z., Abdelmasseh M. (2023). Global burden of chronic respiratory diseases and risk factors, 1990–2019: An update from the Global Burden of Disease Study 2019. eClinicalMedicine.

[3] Oh J., Kim S., Yim Y., Kim M.S., Hay S.I., Shin J.I., Yon D.K. (2026). Global, regional, and national burden of chronic respiratory diseases and impact of the COVID-19 pandemic, 1990–2023: A Global Burden of Disease study. Nat. Med..

[4] Atsou K., Chouaid C., Hejblum G. (2011). Variability of the chronic obstructive pulmonary disease key epidemiological data in Europe: Systematic review. BMC Med..

[5] Raherison C., Girodet P.-O. (2009). Epidemiology of COPD. Eur. Respir. Rev..

[6] Marshall D.C., Al Omari O., Goodall R., Shalhoub J., Adcock I.M., Chung K.F., Salciccioli J.D. (2022). Trends in prevalence, mortality, and disability-adjusted life-years relating to chronic obstructive pulmonary disease in Europe: An observational study of the global burden of disease database, 2001–2019. BMC Pulm. Med..

[7] Ulmeanu R., Fildan A.P., Rajnoveanu R.M., Fira-Mladinescu O., Toma C., Nemes R.M., Tudorache E., Oancea C., Mihaltan F. (2020). Romanian clinical guideline for diagnosis and treatment of COPD. J. Int. Med. Res..

[8] Global Initiative for Asthma (2026). Global Strategy for Asthma Management and Prevention.

[9] Lee W., Nagubadi S., Kryger M.H., Mokhlesi B. (2008). Epidemiology of Obstructive Sleep Apnea: A Population-based Perspective. Expert Rev. Respir. Med..

[10] Alenezi M.A., Alabdulathim S., Alhejaili S.A.M., Al Sheif Z.A.A., Aldossari K.K., Bakhsh J.I., Alharbi F.M., Ahmad A.A.Y., Aloufi R.M., Mushaeb H. (2024). The Association Between Obesity and the Development and Severity of Obstructive Sleep Apnea: A Systematic Review. Cureus.

[11] Buist A.S., McBurnie M.A., Vollmer W.M., Gillespie S., Burney P., Mannino D.M., Menezes A.M., Sullivan S.D., Lee T.A., Weiss K.B. (2007). International variation in the prevalence of COPD (the BOLD Study): A population-based prevalence study. Lancet.

[12] Zein J.G., Erzurum S.C. (2015). Asthma is Different in Women. Curr. Allergy Asthma Rep..

[13] Heinzer R., Vat S., Marques-Vidal P., Marti-Soler H., Andries D., Tobback N., Mooser V., Preisig M., Malhotra A., Waeber G. (2015). Prevalence of sleep-disordered breathing in the general population: The HypnoLaus study. Lancet Respir. Med..

[14] Tobacco Consumption Statistics. https://ec.europa.eu/eurostat/statistics-explained/index.php?title=Tobacco_consumption_statistics.

[15] Laverty A.A., Vardavas C.I., Filippidis F.T. (2021). Prevalence and reasons for use of Heated Tobacco Products (HTP) in Europe: An analysis of Eurobarometer data in 28 countries. Lancet Reg. Health-Eur..

